# Digital crowdsourced intervention to promote HIV testing among MSM in China: study protocol for a cluster randomized controlled trial

**DOI:** 10.1186/s13063-020-04860-8

**Published:** 2020-11-17

**Authors:** Ci Ren, Joseph D. Tucker, Weiming Tang, Xiaorun Tao, Meizhen Liao, Guoyong Wang, Kedi Jiao, Zece Xu, Zhe Zhao, Yu Yan, Yuxi Lin, Chuanxi Li, Lin Wang, Yijun Li, Dianmin Kang, Wei Ma

**Affiliations:** 1 Department of Epidemiology, School of Public Health, Cheeloo College of Medicine, Shandong University, Jinan, 250012 Shandong China; 2 University of North Carolina Chapel Hill Project-China, No. 2 Lujing Road, Guangzhou, 510095 China; 3 Institution for AIDS/STD Control and Prevention, Shandong Center for Disease Control and Prevention, Jinan, 250014 Shandong China

**Keywords:** HIV testing, Men who have sex with men (MSM), Digital, Crowdsourced intervention, Cluster-randomized controlled trial, China

## Abstract

**Background:**

Men who have sex with men (MSM) are an important HIV key population in China. However, HIV testing rates among MSM remain suboptimal. Digital crowdsourced media interventions may be a useful tool to reach this marginalized population. We define digital crowdsourced media as using social media, mobile phone applications, Internet, or other digital approaches to disseminate messages developed from crowdsourcing contests. The proposed cluster randomized controlled trial (RCT) study aims to assess the effectiveness of a digital crowdsourced intervention to increase HIV testing uptake and decrease risky sexual behaviors among Chinese MSM.

**Methods:**

A two-arm, cluster-randomized controlled trial will be implemented in eleven cities (ten clusters) in Shandong Province, China. Targeted study participants will be 250 MSM per arm and 50 participants per cluster. MSM who are 18 years old or above, live in the study city, have not been tested for HIV in the past 3 months, are not living with HIV or have never been tested for HIV, and are willing to provide informed consent will be enrolled. Participants will be recruited through banner advertisements on Blued, the largest gay dating app in China, and in-person at community-based organizations (CBOs). The intervention includes a series of crowdsourced intervention materials (24 images and four short videos about HIV testing and safe sexual behaviors) and HIV self-test services provided by the study team. The intervention was developed through a series of participatory crowdsourcing contests before this study. The self-test kits will be sent to the participants in the intervention group at the 2nd and 3rd follow-ups. Participants will be followed up quarterly during the 12-month period. The primary outcome will be self-reported HIV testing uptake at 12 months. Secondary outcomes will include changes in condomless sex, self-test efficacy, social network engagement, HIV testing social norms, and testing stigma.

**Discussion:**

Innovative approaches to HIV testing among marginalized population are urgently needed. Through this cluster randomized controlled trial, we will evaluate the effectiveness of a digital crowdsourced intervention, improving HIV testing uptake among MSM and providing a resource in related public health fields.

**Trial registration:**

ChiCTR1900024350. Registered on 6 July 2019.

## Background

Chinese MSM still have a high burden of incident HIV infection, suggesting the need for enhanced HIV control strategies. As of October 2019, MSM accounted for 23.0% of newly reported HIV cases in China, increased from 14.7% of new HIV cases in 2011 [1, 2]. Although the Chinese clinical and public health systems have organized many free HIV testing programs, two systematic reviews show that the HIV testing rate among MSM in China remains low [3, 4]. In order to improve the HIV testing rate and reach the first 90 target of the 90-90-90 UNAIDS goals [5], innovative approaches are urgently needed. Digital crowdsourced media may be a useful tool. We define digital as social media, mobile phone applications, Internet, or other approaches that use digital connections.

Digital approaches have been used to promote HIV prevention, especially for key populations like MSM [6–9]. Digital approaches can reach remote participants, limit stigma, provide a platform for anonymity and privacy, and increase community engagement [7, 9–11]. Previous surveys suggested that digital interventions can promote HIV testing and safe sexual behaviors through providing information related to HIV, offering HIV testing services, collecting different creative ideas to design intervention and linking people to the community [10, 12]. In China, the Internet has become a major way to find gay partners among MSM [13, 14]. A cross-sectional survey of 61 cities in China showed that 73.5% of MSM had found recent sex partners online [15], providing a strong foundation for digital interventions. According to the data of MSM national sentinel surveillance in China, only 26.3% men had received an HIV test and know the results in the last year [16]. The high rates of Internet use and low HIV testing rate among MSM in China make digital approaches a useful tool in reaching high-risk populations and building low-cost HIV prevention interventions to promote HIV testing [17, 18].

Crowdsourcing provides an opportunity to develop HIV testing services tailored to meet local community needs while [19, 20]. Crowdsourcing has a group of experts and non-experts working together to solve a common problem and sharing solutions with the public [19, 21]. Crowdsourcing may be useful in health research and has been used in cancer, malaria, and HIV research [22–25]. It has several advantages. First, crowdsourcing provides a platform for the people who have no formal training to make a contribute and enhance community engagement [20, 26–28]. Second, crowdsourcing approaches have been found effective in many trials [24, 29]. Third, crowdsourcing can help to access vulnerable groups that would otherwise be difficult to engage [27, 30]. As a result, crowdsourcing also represents a relatively low-cost method to collect information from a large number of people [20, 24, 28, 31]. However, most crowdsourced interventions have been conducted in large cities with few in smaller cities and less developed areas [29, 32]. Digital crowdsourced media may be a good way to promote HIV testing and healthy sexual behaviors in rural or remote regions.

This study proposal aims to describe the designing of the cluster RCT and to evaluate the efficacy of digital crowdsourced intervention to improve HIV testing uptake among MSM in China.

### Trial aims

The first aim of this trial is to improve HIV testing uptake among MSM in eleven target cities. The study will evaluate the effectiveness of a digital crowdsourced intervention compared to current HIV interventions organized by local centers for disease control and prevention (CDCs). We hypothesize that the digital crowdsourced intervention will be superior in improving HIV testing uptake. The second aim is to compare secondary outcomes (including condom use, social networking, HIV stigma, and others) between the digital crowdsourced intervention arm and the current HIV intervention. We hypothesize that the digital crowdsourced intervention will be superior in promoting safe sexual behaviors.

## Methods

### Design

**Fig. 1:**
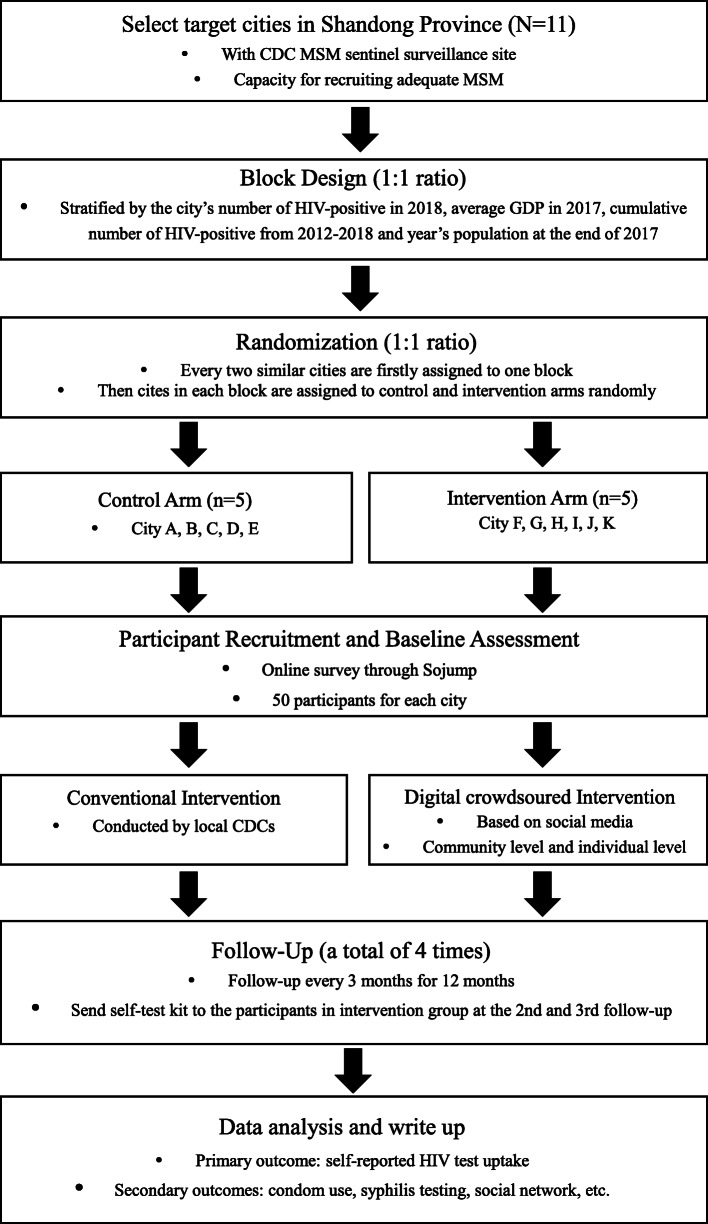
Schematic of crowdsourced intervention implementation

**Fig. 2:**
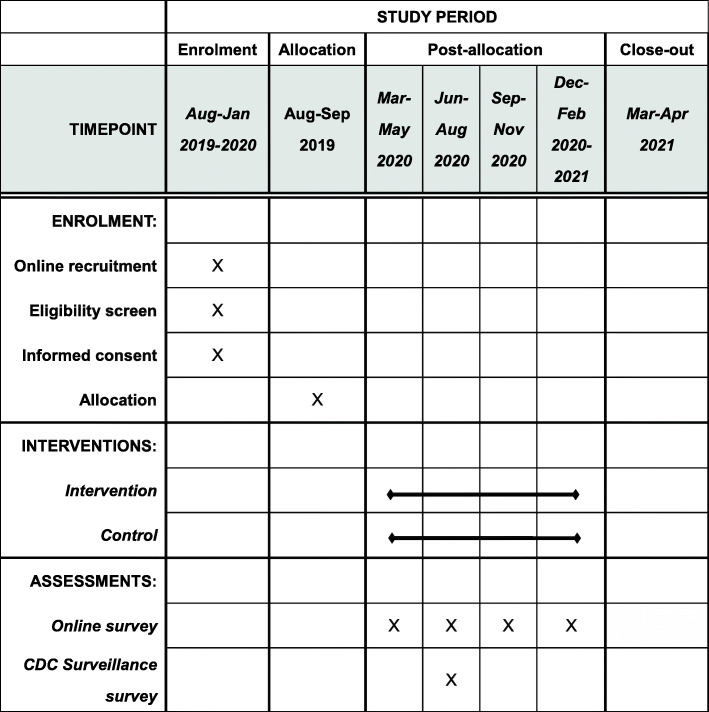
SPIRIT figure of study timeline

As the intervention in our study will involve two levels—the community level and the individual level, and MSM in the same city often communicate with each other, we adopt a cluster-randomized controlled trial with one intervention arm and one control arm (Fig. 1). The cluster unit will be the city. Surveys will be conducted at baseline and every 3 months thereafter (Fig. 2). A total of ten clusters, including City A, City B, City C, City D, City E, City F, City G, City H, City I, City J, and City K from Shandong Province, were chosen based on the following criteria: (1) CDC MSM sentinel surveillance sites and (2) capacity for recruiting sufficient MSM. City J and a nearby City K are in the same cluster because the number of estimated MSM in both cities is small.

### Randomization

We randomly assigned cities in each block (1:1) to either the intervention group or the control group with a block design, stratified by the city’s number of people living with HIV in 2018, average GDP in 2017, cumulative number of HIV-positive from 2012 to 2018 and population at the end of 2017. We have a total of five blocks involving ten clusters: Block 1 consists of City A and City F; Block 2 consists of City B and City G; Block 3 consists of City C, City J, and City K; Block 4 consists of City D and City H; and Block 5 consists of City E and City I. Then, the ten clusters were randomized with five (City A, City B, City C, City D, City E) in the intervention arm and five (City F, City G, City H, City I, City J, City K) in the control arm. Random numbers were generated using SAS 9.4 software. Cities in the intervention arm will implement the digital crowdsourced intervention while cities in the control arm will implement conventional interventions organized by local CDCs. Participants and data analysts will be blinded in this trial.

### Participants and eligibility criteria

Eligibility criteria for participants were as follows: (1) aged at least 18 years, (2) had anal sex with men in the past 1 year, (3) currently living and planning to live in the city for the next 12 months, (4) born biologically male, (5) agree to provide phone number (just for follow up), (6) willing to participate in the follow-up survey every 3 months, and (7) fully understand the facts about the trial with the informed consent. The informed consent form will be shown to the potential participants at the beginning of the online questionnaire. Only those who clicked the “agree” option may be included in the study. Participants living with HIV will be invited to complete the baseline survey but will not be followed.

### Study measures and recruitment

We have built an online questionnaire (see Additional file 1) using Sojump Survey Software. The questionnaire is divided into two parts. Part 1 includes the informed consent form and six items related to study eligibility. Part 2 includes the main items related to this trial. MSM who are determined eligible in the first part proceed to the second section. They will be required to add us as contacts on WeChat (the largest social mobile phone app in China) so that we can establish an online cohort. Participants who do not meet all requirements can scan the QR codes to get 0.14USD (1RMB) on WeChat as an inducement. MSM will be recruited through banner advertisements on Blued, the largest gay dating app in China. CBOs in each study city will also assist with recruitment. In addition to direct recruitment through digital approaches, eligible participants will be invited to refer no more than five friends from their social networks of MSM and can receive a 1.44USD (10RMB) per person as incentive if their friends meet the requirements of this trial. All participants who enroll in the trial will receive a 7.22USD (50RMB) incentive for baseline survey and 7.22USD (50RMB) for each follow-up. Surveys will be conducted at baseline and every 3 months thereafter. Those who complete all five surveys will have an opportunity to win an iPad mini.

All the information in the questionnaire is self-reported. The questionnaire used in MSM surveillance sites run by local CDCs in each city will include additional questions about sexual partners, social media engagement, HIV/syphilis testing, and HIV/syphilis test results (see Additional file 2). Cell phone numbers will be used to link CDC and online survey data sets so the self-report data can be triangulated with CDC surveillance data during the same period.

### Intervention

The intervention includes 24 images and 4 short videos about HIV testing and safe sexual behaviors. We will also send self-test kits to the participants in intervention group with their consent to evaluate the effectiveness of self-test kit in improving HIV testing rate at the 2nd and 3rd follow-ups. Images and videos were developed through a series of participatory crowdsourcing contests before this study. These included a sprint-like designathon that identified digital HIV self-testing as a priority [33] and an open call for images and videos [29]. The 24 images aim to address the importance of HIV testing to improve HIV testing uptake. The purpose of the 4 short videos of 50 s to 1 min in duration is to enhance the awareness of safe sexual behaviors like condom use among MSM and encourage them to take regular HIV testing. The images and videos can be found in Additional file 3. The intervention will be implemented at the individual level through “WeChat”, and at community level through WeChat-group and WeChat-moments (a function on WeChat that people can share their life with friends).

The intervention will be implemented in five clusters (City F, City G, City H, City I, City J, City K) in the intervention arm. For individual-level intervention, the images will first be shown at the end of the baseline questionnaire, and then, we will send one image every 2 weeks and one video every 3 months to the participants via WeChat. We will also send self-test kits to the participants in the intervention group at the 2nd and 3rd follow-ups. As part of the community-level intervention, we will set up several WeChat groups in each city and invite 30–40 participants in each group. Messages about HIV testing and safe sexual behavior from authoritative facilities (hospital, CDCs, CBOs, etc.) will be distributed to these groups and WeChat-moments every 2 weeks. We will encourage members in these groups to talk about HIV through these private groups. We will invite volunteers from local CBOs to join the groups. Team members of this study will participate in discussion if the participants have any question. Participants in other five cities (City A, City B, City C, City D, City E) will receive routine intervention by local CDCs. All concomitant care is acceptable with no restrictions on what participants may seek.

### Follow-up

All participants will receive an online survey at baseline and follow-up surveys will be conducted every 3 months. Each man will participate in a total of five surveys. At the 2nd and 3rd follow-ups, we will send self-test kits to the participants in the intervention group to evaluate the effectiveness of self-test kit in improving HIV testing rate.

### Outcomes

The primary outcome of this study will be the uptake of self-reported HIV testing uptake after 12 months. We have set ten monitoring sentinels in the eleven cities so that the self-reported HIV testing rate can be triangulated with the rate from surveillance data during the same period. In addition, participants who receive the self-test kit are required to send their results to us on WeChat so that we can verify that the self-reported results are consistent with the self-test results. An increase of 20% in testing rate (assuming a proportion of HIV testing of 70% in intervention arm and 50% in control arm) was chosen as the superiority margin. This choice was based on the data of MSM sentinel surveillance collected by CDCs of Shandong Province in last 1 year. Secondary outcomes include condom use, syphilis testing, social network characteristics, and others (see Additional file 4).

### Sample size

A two-arm, cluster-randomized controlled trial will be used. The sample size was calculated based on the primary outcome. We assumed that a digital crowdsourced intervention will be superior to CDC routine intervention in promoting HIV testing rate among MSM who have not been tested in the past 3 months. Assuming the HIV testing rate is 70% in intervention cities and 50% in the control cities, a total of ten clusters (five in intervention group and five in control group), an intraclass correlation coefficient of 0.02 (usually between 0.01 to 0.03), two-sided *α* = 0.05, power = 0.85, and the loss of follow-up is 30%, the total sample size is 500 men (50 for each cluster). The calculation was made using the software PASS 15.

### Timeline

This study will last 17 months. The first 5 months will be the baseline survey. Given the total population of MSM are small in some cities, we wanted to ensure sufficient time for participant recruitment. CDC surveillance surveys will also be conducted in participating cities. The following 12 months will be the intervention phase. The intervention will be implemented in City F, City G, City H, City I, City J, and City K following the cluster RCT design outlined in Fig.2. All the participants in the eleven cities will be surveyed at baseline and every 3 months thereafter. In the 17th month, the last follow-up survey will be implemented.

### Data collection and management

The Sojump Survey Software will be used for data collection and storage. In this study, we will collect information about socio-demographics, sexual behaviors, HIV and syphilis testing behaviors, social network characteristics, and psychological characteristics through an online questionnaire. The online tool and questionnaire were used in our original stepped wedge cluster randomized controlled trial [29]. Socio-demographic characteristics include age, occupation, education level, annual income, marital status, sexual orientation, and sexual orientation disclosure. Behavioral variables consist of sex partners, frequency of sex, condom use, self-reported HIV testing (including both facility-based and self-testing), syphilis testing, and social networking. Psychological characteristics include HIV testing self-efficacy and HIV stigma.

To improve data quality, we have set up a verification mechanism. When a questionnaire has been completed, we will check it first. If it has only one logical error, we will verify with the participant. When there is more than one logical error in the questionnaire, the participant will no longer be included in the study. All questions in our online survey have been set as compulsory questions, which can improve data completeness.

Final survey data completed by all participants will be saved in a secure computer only used for saving data, discarding other privacy issues such as open network environments. Passwords and firewalls will also be used to protect the data.

An internal advisory committee composed of STI experts has been established. The committee will guide us in study design and data analysis and meet regularly to review and assess progress in data collection and research.

### Analysis

The socio-demographic characteristics will be analyzed using descriptive statistics. Comparison in baseline characteristics between the intervention and control arm will be conducted using a chi-squared test adjusted for clustering. The primary outcome of this trial will be the uptake of self-reported HIV testing rate after 12 months. Generalized linear mixed models (GLMM) will be used for the analysis. Intervention status and time will be considered fixed effects, while sites and individual participants with multiple measurements across the four follow-ups will be considered random effects.

The secondary outcomes will include a series of binary variables (continuous variables will be categorized into binary variables), such as number of sexual partners, frequency of anal sex, sex without condoms, frequency of HIV testing (including both facility-based and self-testing), awareness of syphilis status, social network engagement, and others. These will be analyzed similar with the primary outcomes. Furthermore, the interaction effect of intervention between individual level and community level will also be analyzed.

After the 2nd follow-up, we will conduct an interim analysis to evaluate the effectiveness of digital images and videos. After the 4th follow-up, we will also evaluate the effectiveness of digital media and self-test kit using data collected from the 3rd and 4th follow-ups.

The data cleaning, sorting, and analysis will be carried out by team members who do not participate in the randomization and city administration and do not know the treatment allocation. Data analysts were not allowed to modify or delete data. If an outcome is missing for < 15% of participants, analyses will use a complete-case approach. Otherwise, multiple imputation will be used. Participants who miss the follow-up will be included and assumed not achieve the primary and secondary outcomes during the missed follow-up period.

We will also carry out a subgroup analysis based on age (> 30 years old versus ≤ 30 years old) and way of HIV testing (facility-based versus self-testing).

In order to measure contamination, we will conduct per protocol sub analyses to measure whether our results have been affected by contamination.

Results of this study will be distributed to target cities and national stakeholders.

### Adverse events and others

We will capture potential unexpected adverse events via spontaneous self-report. We build one WeChat account for each city. Participants can contact us on WeChat so we can capture questions and potential unexpected adverse events. Additionally, some participants will be recruited through local CBOs. The respective CBO leaders can also help us to collect adverse events.

Adverse events will be fully and truly reported on a regular basis to the study PI.

If any modifications to the protocol are required at any point over the course of the trial, these changes will be communicated to relevant parties by email.

## Discussion

Digital crowdsourcing approach has the potential to reach marginalized populations who face multi-level barriers to care, lower the cost of intervention development, aggregate community wisdom, and spur innovation [20, 26, 29, 31]. Several studies and reviews showed that the digital crowdsourcing can improve HIV services for MSM, promote HIV test uptake, and link men to care [19, 24, 29]. It can be a powerful vehicle for increasing HIV testing uptake and making messages more feasible to implement among MSM. The effectiveness of digital crowdsourcing has been examined in few randomized controlled trials, suggesting the need for more rigorous evaluation. We believe that a large-scale, multi-site cluster RCT is needed to evaluate the effectiveness of digital crowdsourcing.

There are several limitations that should be considered in this study. First, digital approaches may not be able to reach people who do not use online tools frequently. The CBOs in eleven target cities will also assist with recruitment, which may alleviate this problem to some extent. Second, all the information will be self-reported, introducing the potential for bias. However, our online survey allows a high degree of anonymity, we will not collect personal information like name or address, and we will highlight privacy protection methods in the informed consent. In this way, these potential sources of bias may be reduced. Third, MSM will receive 50 RMB when they complete one online questionnaire. Part of them may deliberately falsify their answers in order to obtain the incentive. To reduce this bias, we have established a strict review mechanism. When a questionnaire has been completed, we will check it first. If it has only one logical error, we will verify with the participant. When there is more than one logical error in the questionnaire, the participant will no longer be included in the study. Furthermore, we have an administrator for each city to ensure that recruitment and review work is done in an orderly manner. Triangulation with CDC surveillance site data can facilitate this validation. Fourth, participants from the intervention and control cities may potentially communicate with each other, which may cause study contamination. We will use WeChat and Sojump Survey Software to avoid contamination. Participants will not know the city assignment. Participants will be required to add us as contacts on WeChat so that we can establish an online cohort. WeChat is a closed social app, if you want to talk to someone on WeChat, you have to add him/her as contact first. We will build 10 WeChat accounts for the 10 cities so that participants from different cities cannot contact each other. The Sojump Survey Software was used to collect online questionnaire. In this software, participants will not receive any images or videos when they choose the control arm cites. Furthermore, if a participant did not choose the city he actually lives in, we can find his IP address through the Sojump Software. If his IP address is not consistent with his choice in the questionnaire, the participant will not be included in the study. Although the randomization of this trial is at city level, participants will not be aware of whether they are in the intervention group or the control group. Participants will also be asked whether they have seen the pictures or words which should only been sent to the intervention group in the last follow-up questionnaire.

We anticipate that this digital crowdsourcing intervention will increase HIV testing rate and promote safe sexual behaviors among MSM in China. The study outcomes will help to identify the effectiveness of the intervention. The intervention content can be accessed for free to the public. If successful, we hope that the health institutions will utilize this program as a resource and applied to the HIV prevention or to solve other health issues.

## Trial status

This protocol was registered in chictr.org.cn with ID ChiCTR1900024350, identifier http://www.chictr.org.cn/showproj.aspx?proj=36718. Protocol Version: 4.0, date: 27. March 2020. CONSORT Extension for Cluster Trials 2012 Statement was used to guide the writing of this protocol [34]. Recruitment started on 6 August 2019 and finished on 25 January 2020. At the time of this draft, the recruitment has completed. The original study sites included eight cities. Because we found the progress was slow in several cities during the recruitment, especially in the less developed areas, after waiting a reasonable amount of time and discussing with the advisory committee, we decided to add another three cities in our study. City E and City I are two independent cities which are assigned to one block according to the city’s number of HIV-positive in 2018, average GDP in 2017, cumulative number of HIV-positive from 2012 to 2018, and year’s population at the end of 2017. Random numbers were generated using SAS 9.4 software. Finally, City E was assigned to the control arm, City I was assigned to the intervention arm by randomization. City K is a city nearby City J; in this study, City J and City K are in the same cluster considering the number of estimated MSM in both cities is small. In other words, City J and City K were considered as one city in this trial. After adding three cities to the study, we have a total of ten clusters, five in the intervention group and five in the control group. After re-calculating sample size, the total sample size is 500 men (50 to each cluster).

## Supplementary information

**Additional file 1.** Online survey instrument (English version)

**Additional file 2.** CDC surveillance survey instrument

**Additional file 3. **Intervention materials

**Additional file 4.** Table for secondary outcomes

**Additional file 5.** SPIRIT 2013 Checklist: Recommended items to address in a clinical trial protocol and related documents

## Data Availability

Data is not applicable now. Materials can be found in Additional file 3. People who are interested in the data can contact us after the trial is completed.

## Abbreviations

AIDS: Acquired immune deficiency syndrome
CBO: Community-based organization
CDC: Chinese Center for Disease Control and Prevention
GLMM: Generalized linear mixed models
HIV: Human immunodeficiency virus
LMIC: Low-income and middle-income countries
MSM: Men who have sex with men
QR code: Quick response code
RCT: Randomized controlled trial
STI: Sexually transmitted infection
UNAIDS: The Joint United Nations Programme on HIV and AIDS
