# Supplementary material for: Digital crowdsourced intervention to promote HIV testing among MSM in China: study protocol for a cluster randomized controlled trial
Source: Trials. 2020 Nov 17;21:931. doi: 10.1186/s13063-020-04860-8 (PMC7673095; doi:10.1186/s13063-020-04860-8)
Supplement: Supplementary file 1 — Additional file 1. Online survey instrument (English version) [file 13063_2020_4860_MOESM1_ESM.docx]

**Additional File 1. Online survey instrument (English version)**

**Part I**

**Health survey of Shandong University**

**About this Study:**

Hello, we the research team of Shandong University, thank you very much for participating in our survey!

This questionnaire has many pages, but most of the pages have only 1-2 single-choice questions. Your part in this current survey will last approximately 10-15 minutes. We will send you a red envelope of 50 RMB after you submit the questionnaire.

If you have any questions, please call Ci Ren, the contact person of this project.

Phone number: 17854143882.

**Online Consent Form**

**Title of Study:** Health survey of Shandong University

**IRB study number:** 20190210 **Principal Investigator:** Dr. Wei Ma
Dr. Wei Ma, PHD tutor, School of Public Health, Shandong University, 44 West Wenhua Road, Jinan, Shandong Province, 250012, China. E-mail: [weima@sdu.edu.cn](mailto:weima@sdu.edu.cn).

**What are some general things you should know about this research studies?** You are being asked to participate in a research study. To join this research study is voluntary. You may for whatever reason refuse to join or withdraw your consent to be in the study at any time. Details about this study are discussed below. It is important that you understand this information so that you can make an informed choice about joining this research study.

**What is the purpose of this study?** Innovative approaches to HIV testing promotion campaigns are urgently needed. The current strategy to developing many of these campaigns is to repackage old ideas rather than create new ones. The study aimed to promote HIV testing and safe sex through a series of online activities for young men who have sex with men (MSM) who have high internet use and are willing to participate in online forums.

**How many people will take part in this study?** If you decide to participate in this research study, you will be one of approximately 2000 individuals.

**What will happen if you take part in the study?** Your part in this current survey will last approximately 10-15 minutes. Follow-up surveys will last approximately 5-10 minutes. During this current survey, you will be asked to complete an online questionnaire. The study questionnaires will ask you to provide sociodemographic information as well as details about your sexual health and sexual activity. Upon completion of this initial questionnaire, you will be asked to input your mobile phone number as a means for the research team to prevent duplicate responses, to send reminders, and to distribute rewards for participation. You may be invited to join the WeChat group to participate in online activities (including pushing materials related to HIV prevention, popularizing HIV-related knowledge, etc.) with other MSM. Your information will be kept strictly confidential. A subset of participants will be asked to complete one follow up survey once every three months for a year, consisting of four follow up surveys in total. If you do not respond to the initial follow up request, you will receive a message reminder. To do this, we will also ask you to provide your WeChat number. Your mobile and WeChat number will be strictly confidential and will not be used for any other purposes.

**What are the possible benefits from being in this study?** Research is designed to benefit society by gaining new knowledge. The field of HIV interventions among young MSM in resource-limited settings is in its infancy. The results from this study will help the research team develop an MSM targeted, community-level intervention that will be fielded and evaluated in the Chinese setting. Your participation will also help design better interventions to promote HIV testing among MSM in China.

**How will your privacy be protected?** All data is directly entered into computers as participants complete the questionnaires. Access to the data will be password protected within the server’s firewall. Cookies will not be used in any way to track participant activity. We will delete all your privacy information like phone and WeChat number immediately after you finish all four follow-up surveys.

**Will you receive anything for being in this study? Will it cost anything? Participants will have the opportunity to earn up to 250 RMB WeChat red pocket – it will be distributed as 5 separate 50 RMB WeChat red pocket top-ups and an iPad mini. Participants will receive 50 RMB upon completion of the first questionnaire. We will send some images or texts related to HIV testing and the link of the follow-up survey to some participants in the following one year. Participants will gain another 200 RMB for the 3/6/9/12 month follow up surveys if they are eligible. For those who complete all of the follow up surveys, they will have a chance to win an iPad mini.** There are no costs associated with participating in this research study.

**What if you have questions about this study?** If you have any questions, complaints, or concerns about the research or your participation in the study, feel free to contact Ci Ren (17854143882)

**What if you have questions about your rights as a research participant?** All research on human volunteers is reviewed by a committee that works to protect your rights and welfare. If you have questions or concerns, or if you would like to obtain information or offer input, please contact the Ethics Committee of Public Health in Shandong University at 0531- 88382091.

Q1. If you understand and agree to participate in this research study, please select “Agree” from the options below. We thank you for your participation!

- Agree
- Decline (Skip to End of Survey when finished Q6)

**Q. Basic information**

Q2. How would you describe your assigned sex at birth?

- Male
- Female (Not eligible to take this survey – Skip to end of survey when finished Q6)

Q3. Please enter your age:

____years-old (Not eligible to take this survey if the year < 18 y/o – Skip to End of Survey when finished Q6)

Q4. In the last year, have you ever had anal sex with another man?

- Yes
- No (Not eligible to take this survey – Skip to end of survey when finished Q6)

Q5. When was your most recent HIV test?

- In the last 3 months (Not eligible to take this survey – Skip to end of survey when finished Q6)
- Between the past 3-6 months
- Between the past 6-12 months
- Before the last 12 months
- I have never been tested for HIV

Q6. Will you agree to participate in our follow-up surveys lasting for the next year, occurring once every three months? For every follow-up survey you complete, you will receive a 50 RMB reward.

- Agree
- Decline (Not eligible to take this survey – Skip to End of Survey)

**Part II**

**Sociodemographics**

Q1. Do you live in one of the following cities, and do not have moving plans in the next 1 year?

- Jinan
- Qingdao
- Zibo
- Zaozhuang
- Dongying (Not eligible to take this survey – Skip to End of Survey)
- Yantai (Not eligible to take this survey – Skip to End of Survey)
- Weifang
- Jining
- Taian (Not eligible to take this survey – Skip to End of Survey)
- Weihai
- Rizhao (Not eligible to take this survey – Skip to End of Survey)
- Binzhou
- Dezhou
- Liaocheng
- Linyi (Not eligible to take this survey – Skip to End of Survey)
- Heze
- None of above (Not eligible to take this survey – Skip to End of Survey)

Q2. Please enter your mobile number: _________________________________

Q3. Which kind of hukou are you currently holding?

- Current city’s hukou
- Other urban hukou in the province
- Rural hukou in the province
- Urban hukou outside the province
- Rural hukou outside the province

Q4. How long have you lived in this city? ______years_________months.

Q5. What is your current living situation?

- Live in a house or apartment that I own
- Live in a house or an apartment I rent
- Rent a room or space in someone else's house or apartment
- Stay with someone else for free
- Live at my workplace (on-site shelter, dorm, etc.)
- Live on the street, or stay at more than two different places a week
- Other

Q6. What’s your occupation?

- Student
- Civil servant
- Farmer
- Labor worker (blue collar)
- Office worker (white collar)
- Seller/service staff
- Technician
- Sex worker
- Unemployed
- Other______

Q7. What is your current legal marital status (referring to women)?

- Not married
- Engaged or Married
- Separated or Divorced
- Widowed

Q8. Do you have any child now?

- Yes
- No

Q9. What is the highest level of education that you have completed?

- High school or below (including Zhongzhuan)
- Some college (Dazhuan)
- College/Bachelors
- Masters/PhD or above

Q10. What is your total individual monthly income from all sources?

- Less than 1500 RMB
- Between 1500 and 3000 RMB
- Between 3001 and 5000 RMB
- Between 5001 and 8000 RMB
- Greater than 8000 RMB

Q11. What is your gender identity?

- Male
- Female
- Transgender
- Unsure/Other

Q12. What is your sexual orientation?

- Homosexual
- Bisexual
- Heterosexual
- Unsure/Other

Q13. Have you ever told anyone about your sexuality or sexual history with men (except your sexual partner)?

- Yes
- No (Skip to Q15)

Q14. Have you ever told health-care providers about your sexuality or sexual history with men?

- Yes
- No

**MSM Basic Situation**

*The next set of questions will ask you about your sexual behaviors with other men.*

**Section 1: Stable male sex partners**

Q15. In your lifetime, have you had any stable male sex partners?

- Yes
- No (Skip to Q27)
- *Stable male partners mean male sex partners who maintain a sexual relationship with you more than 3 months (﹥3 months), including boyfriends and/or regular male sex partners.*

Q16. How old were you when you had anal intercourse with your stable male sex partner for the first time?

________years old *(Number input)*

Q17. The first time you had sex with your stable male sex partner, was it consensual?

- Yes
- No
- *Our definition of consensual is that you were interested in having sex with him, but that you felt like you could have refused or stopped it if you wanted to.*

Q18. In the last 3 months, have you had any stable male sex partners?

- Yes
- No (Skip to Q25)

Q19. In the last 3 months, how many different stable male sex partners have you had?

_________ stable male sex partners *(Number input) (Must be >=1)*

Q20. Where did you mainly meet with your stable male sexual partner(s)? (Select all that apply)

- Pub, disco, tearoom, or club
- Spa or bath house, sauna, foot or body massage parlor
- Park, public restroom, public lawn
- Website
- Social media
- Through friends
- Other

Q21. In the past 3 months, have you had anal sex with your stable male sex partner(s)?

- Yes
- No (Skip to Q25)

Q22. In the past 3 months, when you had anal sex with your stable partner(s), what role did you assume?

- Always insertive (always 1)
- Mostly insertive (mostly 1)
- Both insertive and receptive in similar amounts (Both 1 and 0 in similar amounts)
- Mostly receptive (mostly 0)
- Always receptive (always 0)

Q23. In the last 3 months, approximately how many different stable male sexual partners did you have anal sex with?

_________stable male sex partners *(Number input) (Must be >=1)*

Q24. In the last 3 months, when you had anal sex with your stable male sex partner, how frequently did you use condoms?

- 0% condom use (Skip to Q26)
- Less than 50% condom use
- More than 50% condom use
- 100% condom use

Q25. In your last sexual intercourse with your stable male partner, did you or your sex partner use condoms?

- Yes
- No

Q26. In your lifetime, have you had stable male sex partner(s) living with HIV positive?

- Yes
- No
- I do not know

**Section 2. Casual male sex partners**

Q27. In your lifetime, have you had casual male sex partners?

- Yes
- No (Skip to Q42)
- *Casual male sex partners mean male sex partners who maintain a sexual relationship with you equal to or less than 3 months, including commercial sex partners (≤3 months).*

Q28. How old were you when you had anal intercourse with your casual male sex partner for the first time?

________years old *(Number input)*

Q29. The first time you had sex with your casual male sex partner, was it consensual?

- Yes
- No
- *Our definition of consensual is that you were interested in having sex with him, but that you felt like you could have refused or stopped it if you wanted to.*

Q30. In the last 3 months, have you had casual male sex partners?

- Yes
- No (Skip to Q38)

Q31. In the last 3 months, how many casual male sex partners have you had?

_______________ *(Number input)*

Q32. Where did you meet your casual male sex partners?

- Pub, disco, tearoom, or club
- Spa or bath house, sauna, foot or body massage parlor
- Park, public restroom, public lawn
- Website
- Social media
- Through friends
- Other

Q33. In the last 3 months, have you had anal sex with your casual sex partner(s)?

- Yes
- No (Skip to Q38)

Q34. In the last 3 months, when you had anal sex with your casual male sex partners, what role did you assume?

- Always insertive (always 1)
- Mostly insertive (mostly 1)
- Both insertive and receptive in similar amounts (Both 1 and 0 in similar amounts)
- Mostly receptive (mostly 0)
- Always receptive (always 0)

Q35. In the last 3 months, approximately how many different casual male sexual partners did you have anal sex with?

_________casual male sex partners *(Number input) (Must be >=1)*

Q36. In the last 3 months, when you had anal sex with your casual male sex partner, how frequently did you use condoms?

- 0% condom use (Skip to Q38)
- Less than 50% condom use
- More than 50% condom use
- 100% condom use

Q37. In your last sexual intercourse with your casual male partner, did you or your sex partner use condoms?

- Yes
- No

Q38. Of your casual male sex partners, how many partners were offered money, gifts or drugs to have sex with you?

______ (*Number input*)

Q39. Of your casual male sex partners, how many partners offered money, gifts, or drugs to have sex with you?

______ (*Number input*)

Q40. In your lifetime, have you had casual male sex partner(s) living with HIV positive?

- Yes
- No
- I do not know

Q41. Who was your partner in your last sexual intercourse experience?

- Stable partner (Including boyfriend and regular sexual partner)
- Casual partner (Including irregular sexual partner and commercial sexual partner)

**HIV Testing Behavior**

*The next set of questions will ask about your practices and attitudes in regard to HIV testing.*

Q42. Have you ever been tested for HIV (including both facility-based and self-testing)?

- Yes
- No (Skip to Q66)

Q43. Where did you take the HIV testing during your last test?

- CDC (Skip to Q45)
- Hospital (Skip to Q45)
- Community organization (Skip to Q45)
- The site for blood donation (Skip to Q45)
- Last test was a self-test

Q44. Have you ever been tested for HIV in the hospital, CDC or CBO?

- Yes
- No (Skip to Q55)

*The next set of questions will ask about your practices and attitudes in regards to HIV testing (This is facility-based HIV testing, NOT HIV self-testing).*

Q45. What was the result of your first HIV test (facility-based testing)?

- HIV positive/infected
- HIV negative/uninfected
- I never got my test results
- *Facility-based testing refers to testing in hospitals, CDC, CBO, etc., NOT HIV self- testing.*

Q46. In the past year, how many times have you had a HIV facility-based testing?

_____________________*(Number input)*

Q47. Of these HIV testings in the past year, how many times have you received the results? (Either positive or negative would count)

___________________*(Number input)*

Q48. Of these HIV testings in the past year, how many times did you receive post-test counseling?

___________________*(Number input)*

Q49. What was the result of your most recent HIV test?

- HIV positive/infected
- HIV negative/uninfected (Skip to Q51)
- I never got my test results (Skip to Q52)

Q50. Did you receive a confirmatory Western Blot test?

- Yes
- No
- *Western Blot test means a round of testing to confirm the final result after the initial HIV testing.*

Q51. Did you notify your most recent stable male partner about your most recent HIV test result?

- Yes
- No
- I do not have a stable male partner

Q52. Who was with you when you most recently tested for HIV? (Select all that apply)

- No one, I was alone
- Partner
- Friend who is HIV negative or unknown
- Friend who is HIV positive
- Parent/family

Q53. Did someone else (partner, boss, friend, or others) force you to take an HIV test (facility-based test)?

- Yes
- No

Q54. Has HIV testing (facility-based test) led to a violent confrontation (physical assault)?

- Yes
- No

**HIV Self-Testing**

*The next set of questions will ask about your HIV and STI testing and results. Self-testing refers to you administer the test yourself and interpreting results.*

Q55. Have you ever taken an HIV self-test?

- Yes
- No (Skip to Q66)

Q56. In the past year, how many times have you had a HIV self testing?

_____________________*(Number input)*

Q57. Of these HIV testings in the past year, how many times have you received the results? (Either positive or negative would count)

___________________*(Number input)*

Q58. Have you ever been administered (given) an HIV self-test from other people?

- Yes
- No

Q59. Did someone else (partner, boss, friend, or other) force you to take an HIV self-test?

- Yes
- No

Q60. Was your HIV self-test the first time you ever tested for HIV?

- Yes
- No

Q61. Who was with you when you last took an HIV self-test? (Select all that apply)

- No one, I was alone
- Partner
- Friend who is HIV negative or unknown
- Friend who is HIV positive
- Parent/family

Q62. Has your HIV self-test led to a violent confrontation (physical assault)?

- Yes
- No

Q63. Did you have a positive result in any of your HIV self-test(s)?

- Yes
- No (Skip to Q65)

Q64. Did you confirm your positive HIV self-test result at the CDC or hospital?

- Yes
- No

Q65. Did you receive post self-test counseling?

- Yes
- No

**Testing Norms and Self-Efficacy**

*The following 12 questions will ask about your attitudes toward HIV testing. They ask for your honest opinions, so there are no right or wrong answers.*

Q66. Most gay men who want to get tested but are afraid to get tested.

- Strongly agree
- Agree
- Disagree
- Strongly disagree

Q67. Most gay men who get tested do not want others to find out they were tested.

- Strongly agree
- Agree
- Disagree
- Strongly disagree

Q68. Most gay men want to get tested for HIV.

- Strongly agree
- Agree
- Disagree
- Strongly disagree

Q69. Most gay men who want to get tested will tell their partners they want to get tested.

- Strongly agree
- Agree
- Disagree
- Strongly disagree

Q70. Most gay men have been tested for HIV.

- Strongly agree
- Agree
- Disagree
- Strongly disagree

Q71. Most gay men get tested for HIV only if they are sick or feel uncomfortable.

- Strongly agree
- Agree
- Disagree
- Strongly disagree

Q72. You would feel comfortable discussing HIV testing with a potential partner.

- Strongly agree
- Agree
- Disagree
- Strongly disagree

Q73. You feel confident that you could refuse to have sex with a partner who did not want to undergo HIV testing.

- Strongly agree
- Agree
- Disagree
- Strongly disagree

Q74. You feel confident that you could persuade your partner to undergo HIV testing.

- Strongly agree
- Agree
- Disagree
- Strongly disagree

Q75. You can have an HIV test if you wish.

- Strongly agree
- Agree
- Disagree
- Strongly disagree

Q76. You will have an HIV test even if you are afraid to know the results.

- Strongly agree
- Agree
- Disagree
- Strongly disagree

Q77. You have confidence that you will undergo HIV testing regularly.

- Strongly agree
- Agree
- Disagree
- Strongly disagree

Q78. In the last twelve months, which of the following services did you receive:

- Condom distribution
- Lubricant distribution
- Peer Education
- STD Diagnosis or Treatment
- HIV counseling or Testing
- AIDS/STD Materials (pamphlets, etc.)
- Medical treatment (methadone, ART, etc.)
- Needle exchange
- None of the above

**Syphilis Testing**

Q79. Have you ever been tested for syphilis?

- Yes
- No (Skip to Q82)

Q80. Have you been tested for syphilis in the past 3 months?

- Yes
- No

Q81. Has a doctor ever told you that you had syphilis?

- Yes
- No

**Social media**

*This section asks you about your experiences with activities related to HIV testing online or using social media (Wechat, Weibo, QQ, mobile Apps, etc.).*

Q82. Have you ever looked online for information about HIV testing?

- Yes
- No (Skip to Q86)

Q83. How frequently did you look online for information about HIV testing?

- Once a year at most
- Once every six months
- Once every three months
- Monthly
- Once a week at least

*Please try to recall the LAST time you searched online for information on HIV testing:*

Q84. Did you intend to: (Select all the choices that apply to you)

- Search about the process involved with HIV testing
- Find where to receive an HIV test
- Get more information and knowledge about HIV testing
- Talk to other people about HIV testing
- Other________

Q85. Have you used the following platforms to start searching? (Select all the choices that apply to you)

- At a search engine (like Baidu, Sougou)
- At a site that specializes in HIV testing information (like Gztz, Danlan)_
- The official website of local CDC
- At a more general Q&A site that contains information on all kinds of topics (like Baidu Zhidao, Aiwen, Zhihu)
- At a social network site (like Wechat, Weibo, QQ)
- At a mobile App (like Blued, Jackd, Grindr, Aloha)
- Other________

Q86. Have you ever seen anything related to HIV testing on Weibo, Wechat, QQ messages or mobile Apps?

- Yes
- No (Skip to Q88)

Q87. In the last 3 months, have you seen anything related to HIV testing on Weibo, Wechat, QQ messages or mobile Apps?

- Yes
- No

**Anticipated HIV Stigma**

*Please consider the following statements and indicate to what extent you agree or disagree with them. They ask for your honest opinions, so there are no right or wrong answers.*

Q88. If I had HIV, I’d worry about people discriminating against me.

- Strongly Agree
- Agree
- Disagree
- Strongly Disagree

Q89. If I got infected with HIV no one would date or become involved with me.

- Strongly Agree
- Agree
- Disagree
- Strongly Disagree

Q90. If I got infected with HIV, no one would want to have sex with me.

- Strongly Agree
- Agree
- Disagree
- Strongly Disagree

Q91. If I got infected with HIV, I would work hard to keep my HIV status a secret.

- Strongly Agree
- Agree
- Disagree
- Strongly Disagree

Q92. Upon learning I contracted HIV, I would feel set apart and isolated from the rest of the world.

- Strongly Agree
- Agree
- Disagree
- Strongly Disagree

Q93. If I got infected with HIV, I would feel I was not as good a person as others.

- Strongly Agree
- Agree
- Disagree
- Strongly Disagree

Q94. I would never feel ashamed of getting HIV.

- Strongly Agree
- Agree
- Disagree
- Strongly Disagree

Q95. Will you be willing to participate in a men's health project related to you in the future? (such as volunteering, providing advice and ideas, helping to organize activities in this study, etc.)

- Yes
- No

**Intervention page [ONLY TO WF, ZB, JN, LC, QD]**

**Thank you very much! You have finished all the questions now. Please view this picture for 15s, and then you can fill out your information for receiving the rewards.**


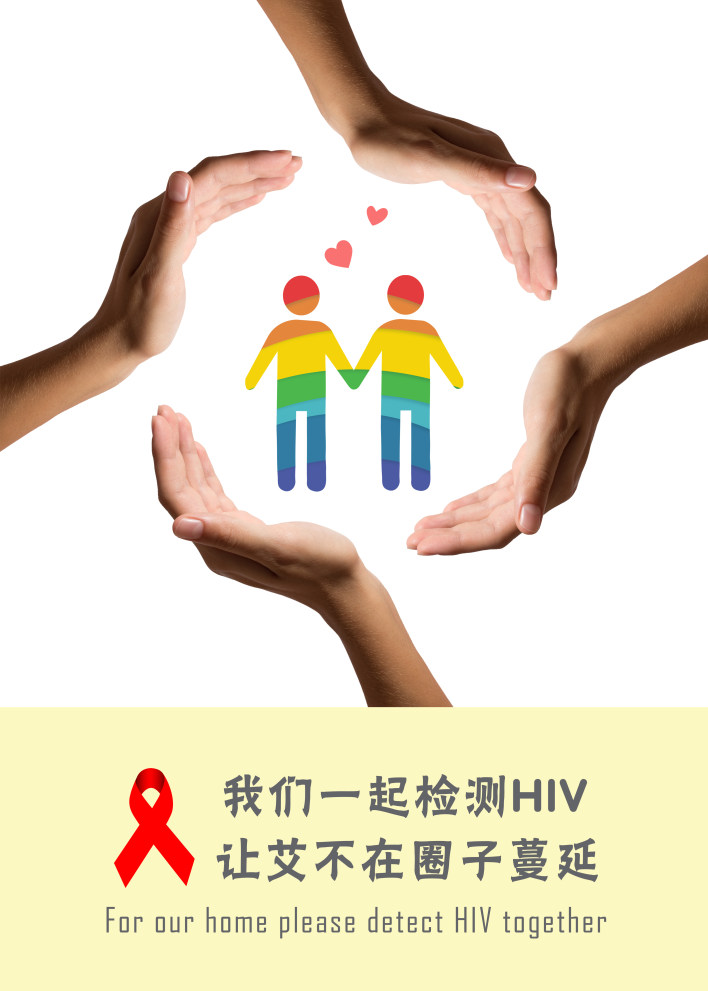


**Ending section**

Please scan the QR code below to add our WeChat named “Health Survey of Shandong University” to get reward. Please note your phone number when you add us. We will not share your personal information to anyone and will not contact you except for the situation of follow up notifications and reward deliveries. After we finish all the follow up surveys, your contact will be deleted.

**[QR code]**

***PS: Different cities have different QR codes. Which QR code will be showed here decided by the Question 1 (Q1) in Part II. This paragraph is not included in the actual questionnaire.***

Please input our WeChat ID:

_________________________________ *(WeChat number input)*

Thanks! You have finished our survey. In the following year, you will receive up to 4 follow up surveys (one about every three months). Each time you complete a survey, you will receive 50 RMB. If you complete all four surveys, you will have a chance to win the latest iPad mini. We hope you can add our WeChat account to receive follow up notifications and rewards.

Please be assured that all your personal information will be protected by a password secured phone. Only staff who would send out the reminder and reward could see your account. We will not share your personal information to anyone and will not contact you except for the situation of follow up notifications and reward deliveries. After we finish all the follow up surveys, your contact will be deleted.
