# Supplementary material for: Digital crowdsourced intervention to promote HIV testing among MSM in China: study protocol for a cluster randomized controlled trial
Source: Trials. 2020 Nov 17;21:931. doi: 10.1186/s13063-020-04860-8 (PMC7673095; doi:10.1186/s13063-020-04860-8)
Supplement: Supplementary file 2 — Additional file 2. CDC surveillance survey instrument [file 13063_2020_4860_MOESM2_ESM.docx]

**Additional File 2. CDC surveillance survey instrument**

**CDC Surveillance Survey Questions**

A1. In the past 6 months, have you had anal sex with man?

- Yes
- No (Skip to B1)

A2. In the past 6 months, who was your main sexual partner?

- Business partner (s)
- Casual partner (s)
- Stable partner (s) (do not include your spouse)
- Spouse

A3. In the past 6 months, have you had sex with more than one man at the same time?

- Yes
- No (Skip to B1)

A4. In the last 6 months, when you had sex with more than one man at the same time, how frequently did you use condoms?

- Never
- Sometimes
- Use condoms every time, but not individually (每次使用但非一人一套)
- Use condoms every time and individually (每次使用一人一套)

B1. In your lifetime, have you ever used drugs (Heroin, Methamphetamine, Magu, Zero Capsule / G Liquid, Rush, K powder, ecstasy, etc.)?

- Yes
- No (Skip to C1)

B2. What kind of drugs do you currently use? (Multiple choice)

- Heroin
- Cocaine
- Opium
- Marijuana
- Morphine
- Methamphetamine
- Du Lingding
- K powder (ketamine)
- Ecstasy
- MaGu
- Rush
- Zero capsule / G spot liquid
- Other____________

B3. In the past 6 months, how often have you used the above drugs?

- Many times / day
- 1 time / day
- 3-5 times / week
- 1-2 times / week
- 1-2 times / month
- Occasionally 1-2 times

B4. In the past 6 months, where have you been using these drugs? (Multiple choice)

- Home
- Friends' house
- Hotel / Guesthouse
- Ballroom / Nightclub
- Bath Center
- Tea Room / Clubhouse
- Office
- Other______________

C1. Excluding this visit to the CDC, in the past year, how often have you received an HIV test?

- Never
- Once
- about twice per year or more frequent
- Did not receive an HIV test in the past year, but have received HIV test before

C2. Excluding this visit to the CDC, in the past year, how often have you received a syphilis test?

- Never
- Once
- about twice per year or more frequent
- Did not receive an HIV test in the past year, but have received HIV test before

C3. In the past three months, have you seen any videos to promote HIV testing?

- Yes
- No

C4. Have you had an HIV Test in the past three months?

- Yes
- No

C5. Have you ever conducted an HIV Self-Testing in the last two years? [HIV self-testing refers to you administering the test yourself and interpreting results]

- Once every two years
- Once a year
- Once every six months
- Once every three months
- Monthly
- Never

**Engagement in Health Promotion Campaigns**

D1. Are you aware of any ongoing community events promoting HIV testing among MSM?

- Yes
- No (Skip to D3)

D2. Have you ever helped organize a testing and/or awareness campaign that promoted HIV testing among MSM?

- Yes
- No

D3. Have you ever volunteered at a health clinic or other location that provided HIV testing services among MSM?

- Yes
- No

D4. Have you ever encouraged someone else to get tested for HIV?

- Yes
- No

D5. Have you ever accompanied a friend or partner to a testing facility to get tested for HIV? [Testing facilities refers to any CDC authorized testing facilities]

- Yes
- No

D6. Have you ever looked online for information about HIV testing?

- Yes
- No (Skip to D9)

D7. Within the past two years, how frequently did you look online for information about HIV testing?

- Once every two years
- Once a year
- Once every six months
- Once every three months
- Monthly
- Weekly
- Daily

D8. When you looked for HIV testing information online, have you used the following platforms? (**Select all that apply**)

- Daily Search engine such as Baidu, haosou, sougou
- Sites that specializes in HIV testing information, like GZTZ, danlan, Shandongtongzhi
- More general sites like baidu zhidao, aiwen, zhihu
- Social network platforms such as WeChat, weibo, QQ, Blued
- Other_______________

D9. In the last 3 months, have you received, liked, posted, followed, discussed, shared, commented anything related to HIV testing on Weibo, Wechat, QQ messages or mobile Apps?

- Yes
- No (Skip to E1)

D10. Please specify whether you have conducted the following behaviors on Weibo, WeChat, QQ messages or mobile Apps:

| Received information about HIV testing | - Yes | - No |
| --- | --- | --- |
| Liked information about HIV testing | - Yes | - No |
| Followed subscription account about HIV testing | - Yes | - No |
| Posted information about HIV testing | - Yes | - No |
| Shared information about HIV testing | - Yes | - No |
| Commented about HIV testing | - Yes | - No |
| Privately discussed about HIV testing | - Yes | - No |
| Discussed in groups about HIV testing | - Yes | - No |

E1. In your lifetime, did someone else (partner, friend, or others) force you to have sex?

- Yes
- No

E2. In the last year, have you been forced to have sex by someone (partner, friend, or others) else?

- Yes
- No

E3. In the last year, have you suffered physical or mental violence from your partner?

- Yes
- No
