# Supplementary figures and images for: Digital crowdsourced intervention to promote HIV testing among MSM in China: study protocol for a cluster randomized controlled trial
Source: Trials. 2020 Nov 17;21:931. doi: 10.1186/s13063-020-04860-8 (PMC7673095; doi:10.1186/s13063-020-04860-8)

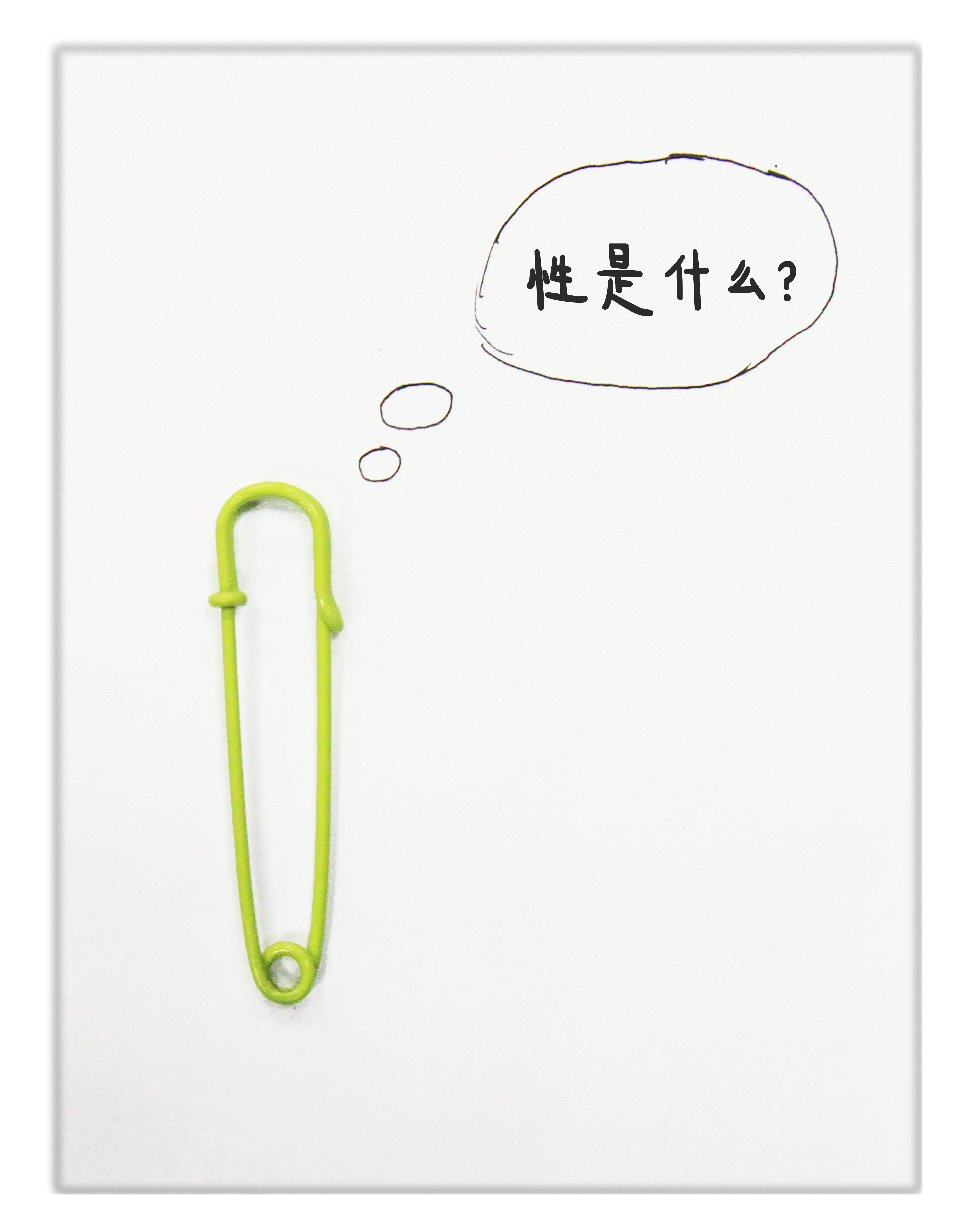

Supplement: Supplementary file 3 — Additional file 3. Intervention materials [file 13063_2020_4860_MOESM3_ESM.zip › Additional file 3/Intervention materials/Images/Image 1/1.jpeg]

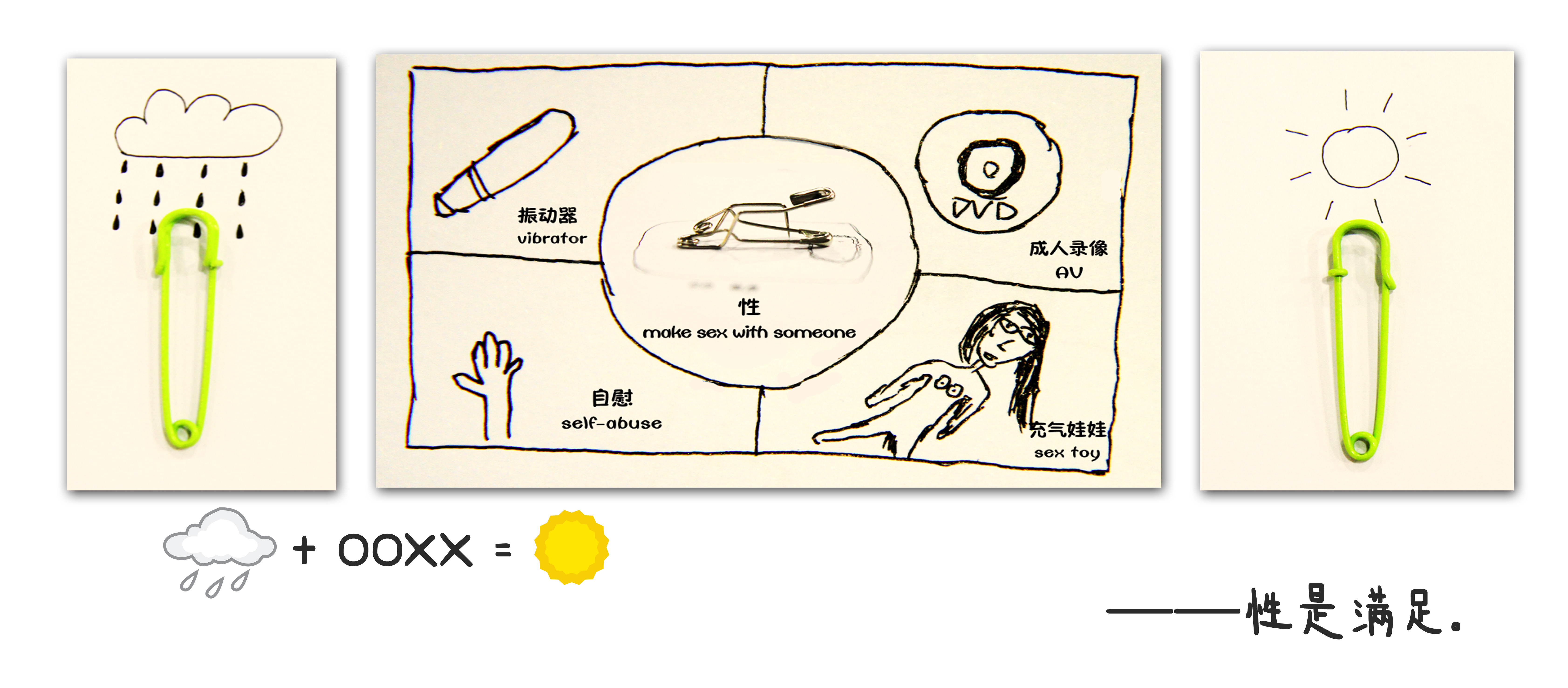

Supplement: Supplementary file 3 — Additional file 3. Intervention materials [file 13063_2020_4860_MOESM3_ESM.zip › Additional file 3/Intervention materials/Images/Image 1/2.jpeg]

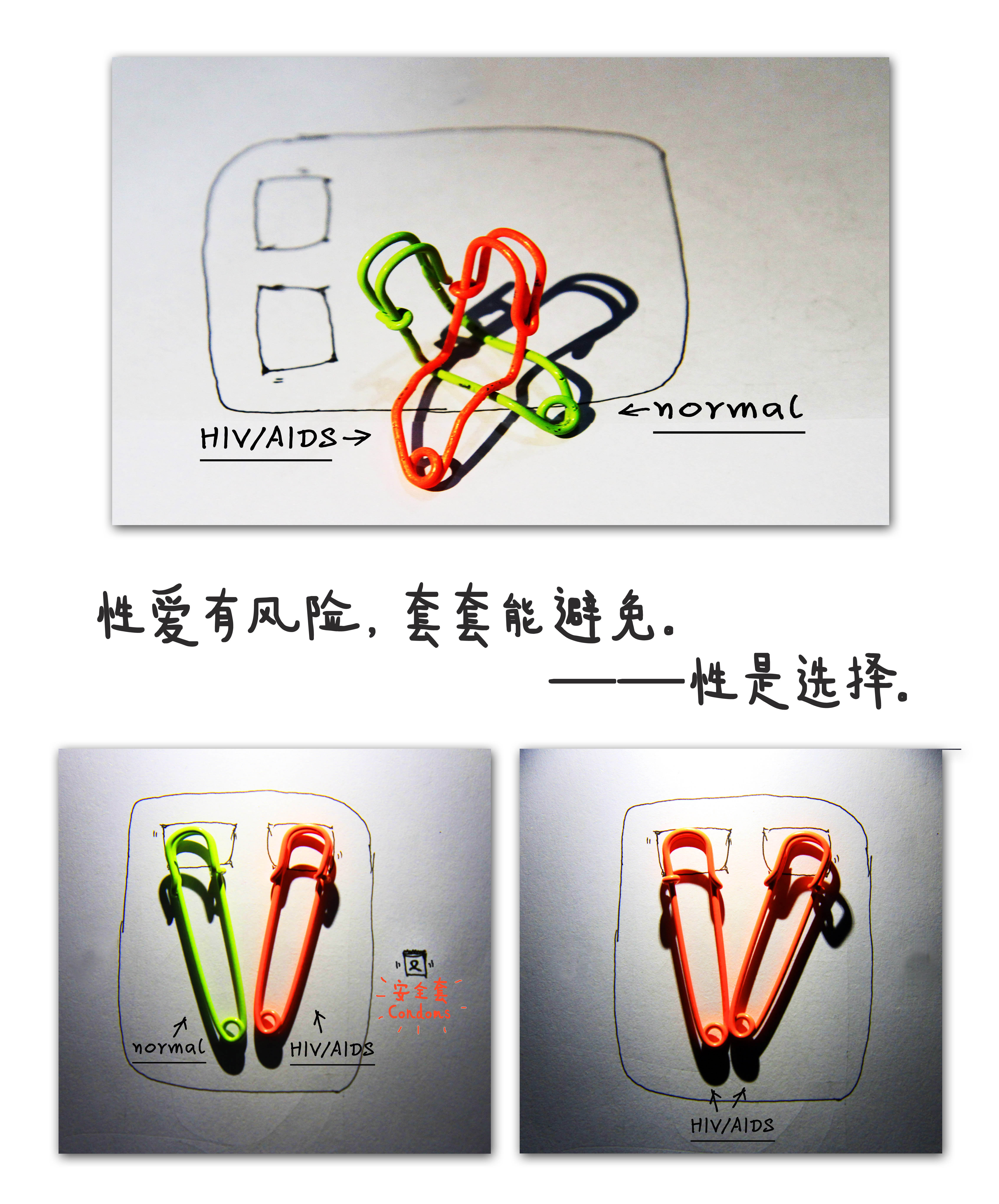

Supplement: Supplementary file 3 — Additional file 3. Intervention materials [file 13063_2020_4860_MOESM3_ESM.zip › Additional file 3/Intervention materials/Images/Image 1/3.jpg]

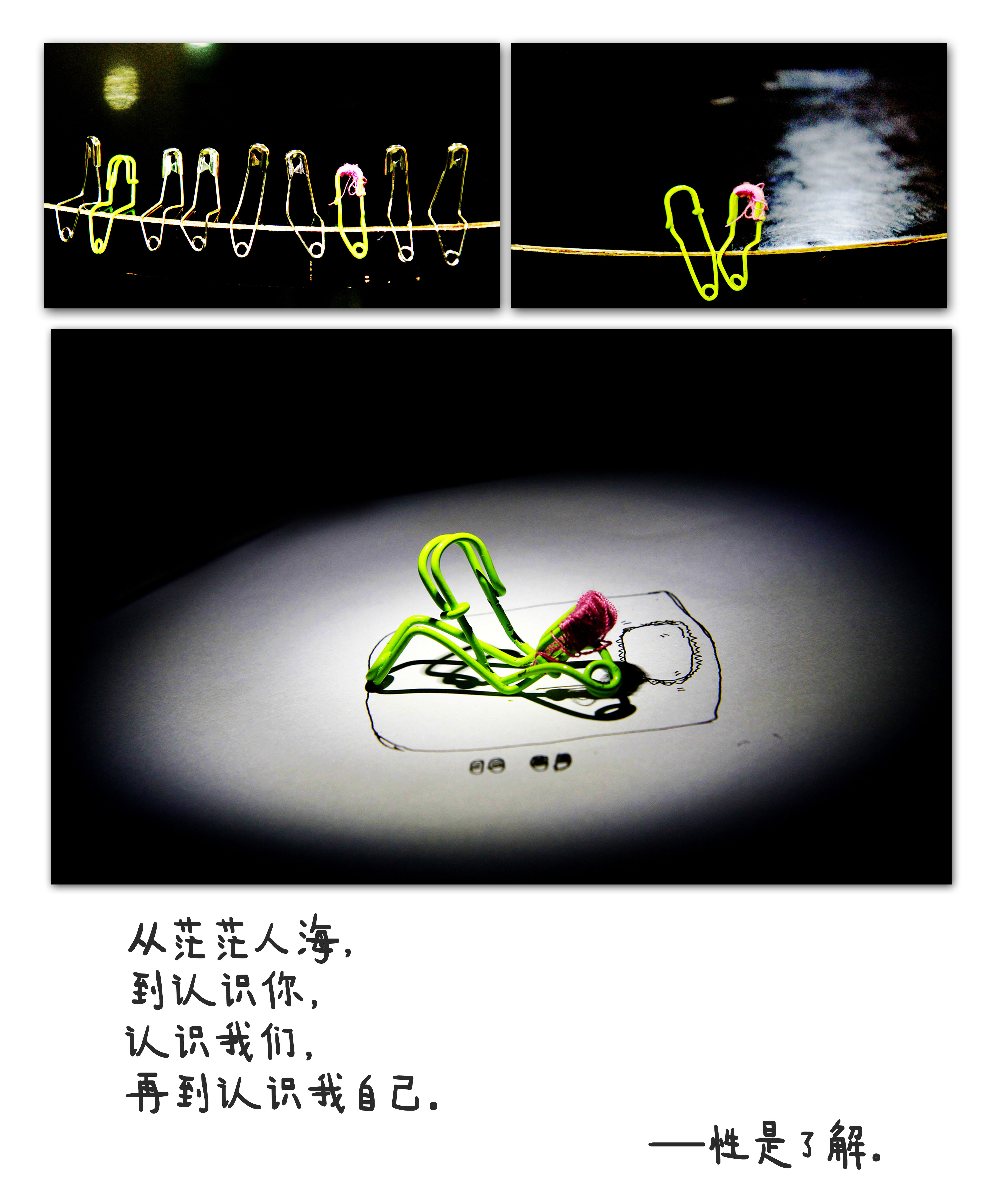

Supplement: Supplementary file 3 — Additional file 3. Intervention materials [file 13063_2020_4860_MOESM3_ESM.zip › Additional file 3/Intervention materials/Images/Image 1/4.jpg]

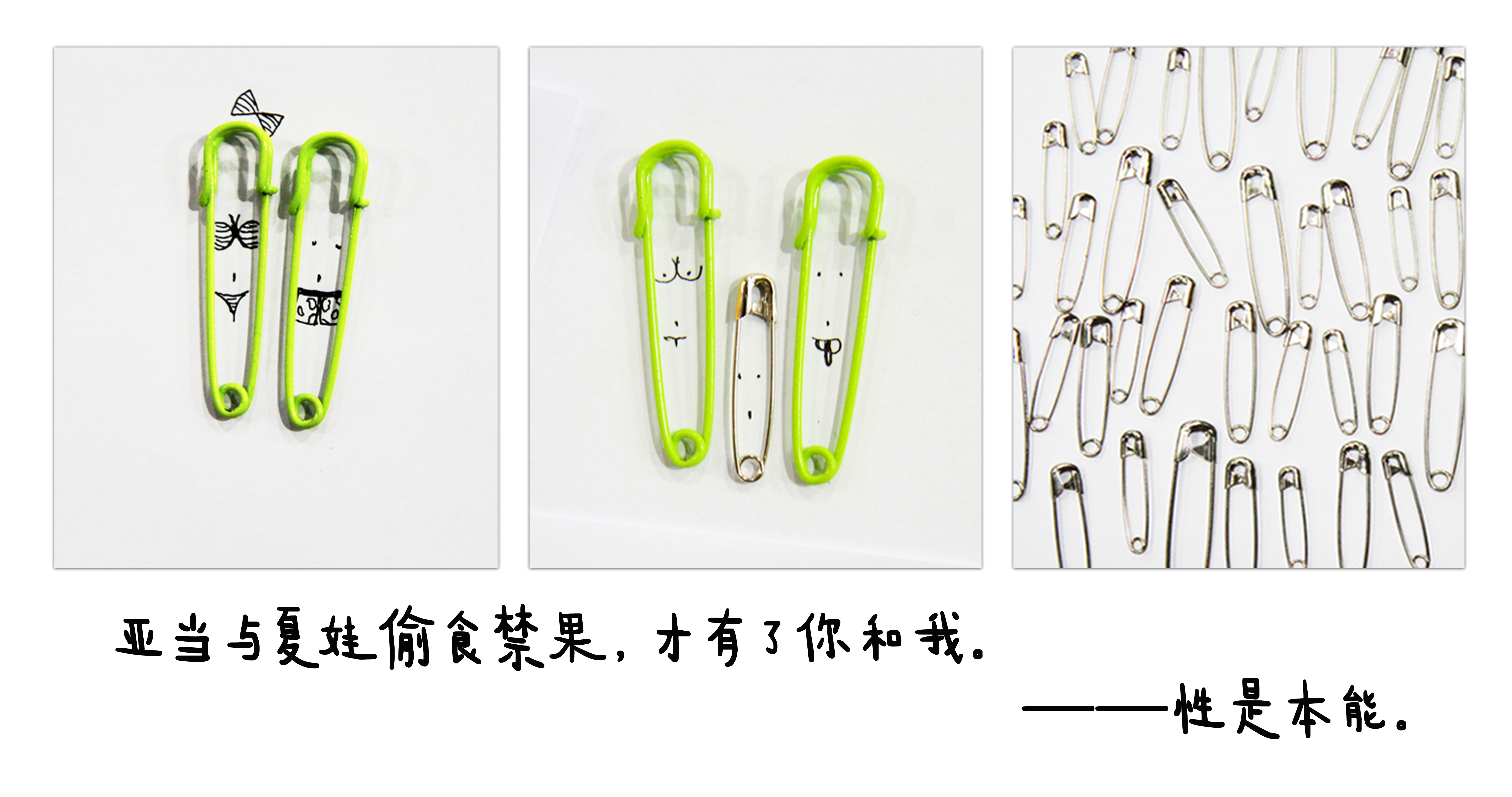

Supplement: Supplementary file 3 — Additional file 3. Intervention materials [file 13063_2020_4860_MOESM3_ESM.zip › Additional file 3/Intervention materials/Images/Image 1/5.jpeg]

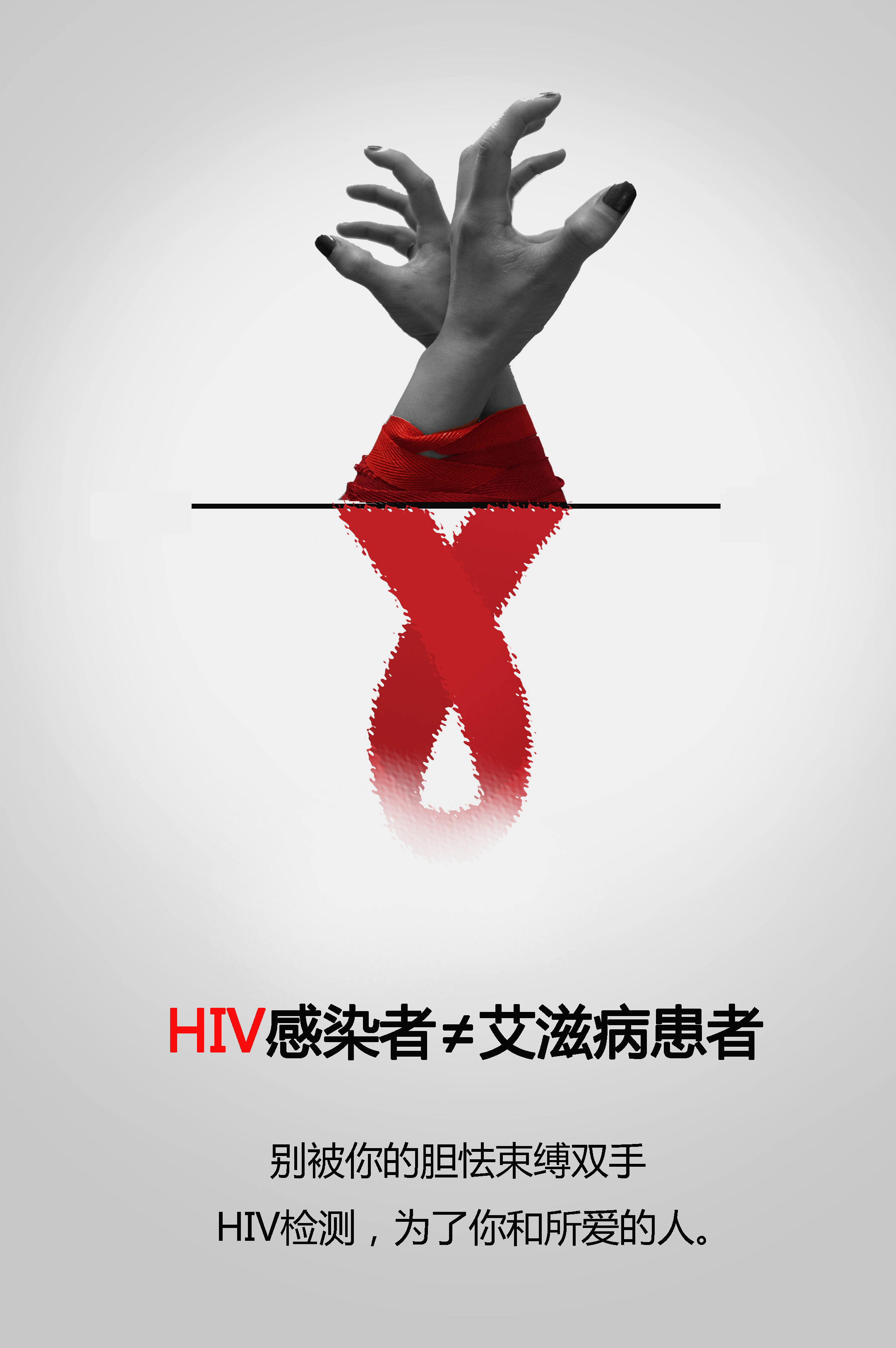

Supplement: Supplementary file 3 — Additional file 3. Intervention materials [file 13063_2020_4860_MOESM3_ESM.zip › Additional file 3/Intervention materials/Images/Image 10.jpg]

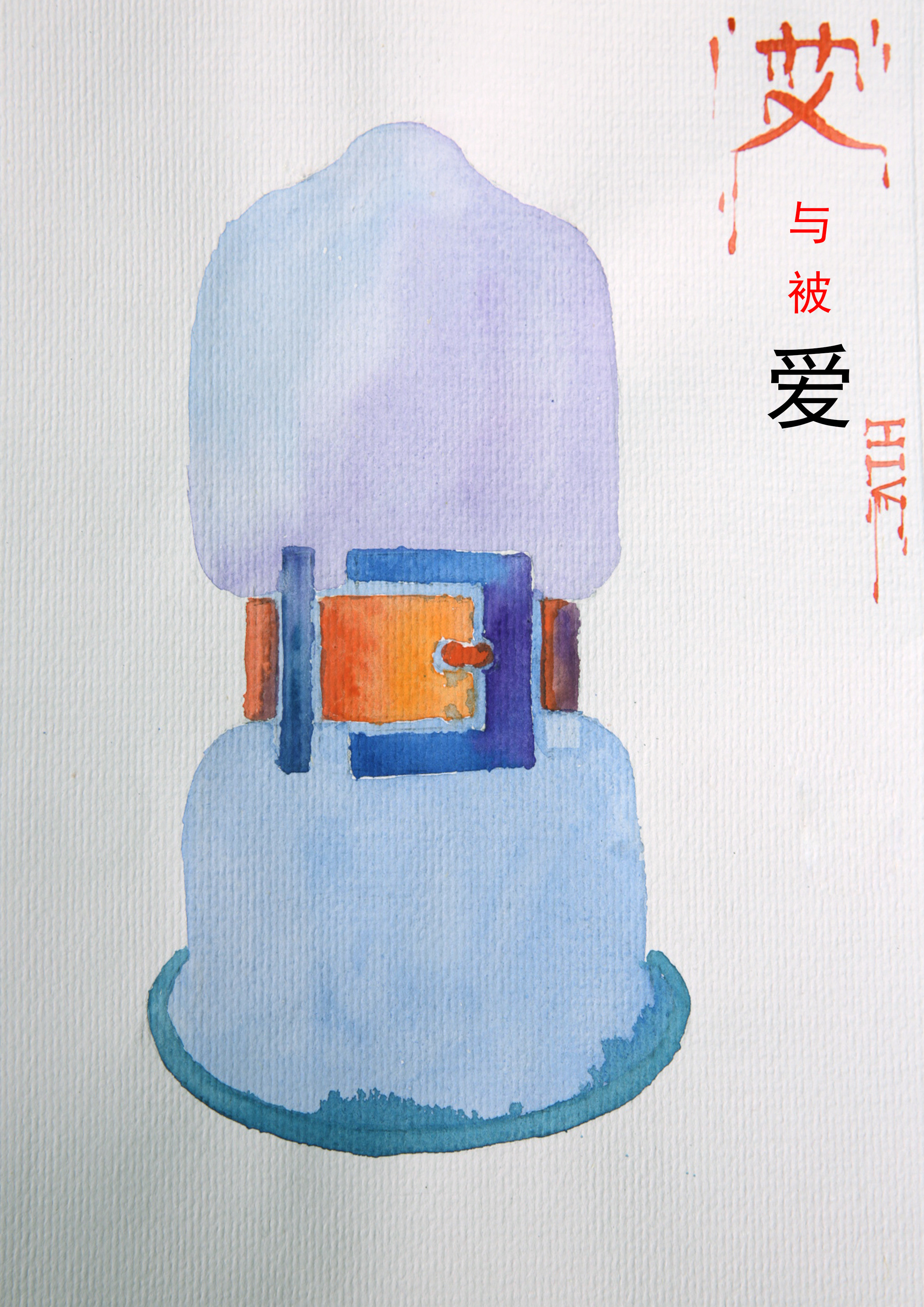

Supplement: Supplementary file 3 — Additional file 3. Intervention materials [file 13063_2020_4860_MOESM3_ESM.zip › Additional file 3/Intervention materials/Images/Image 11.jpg]

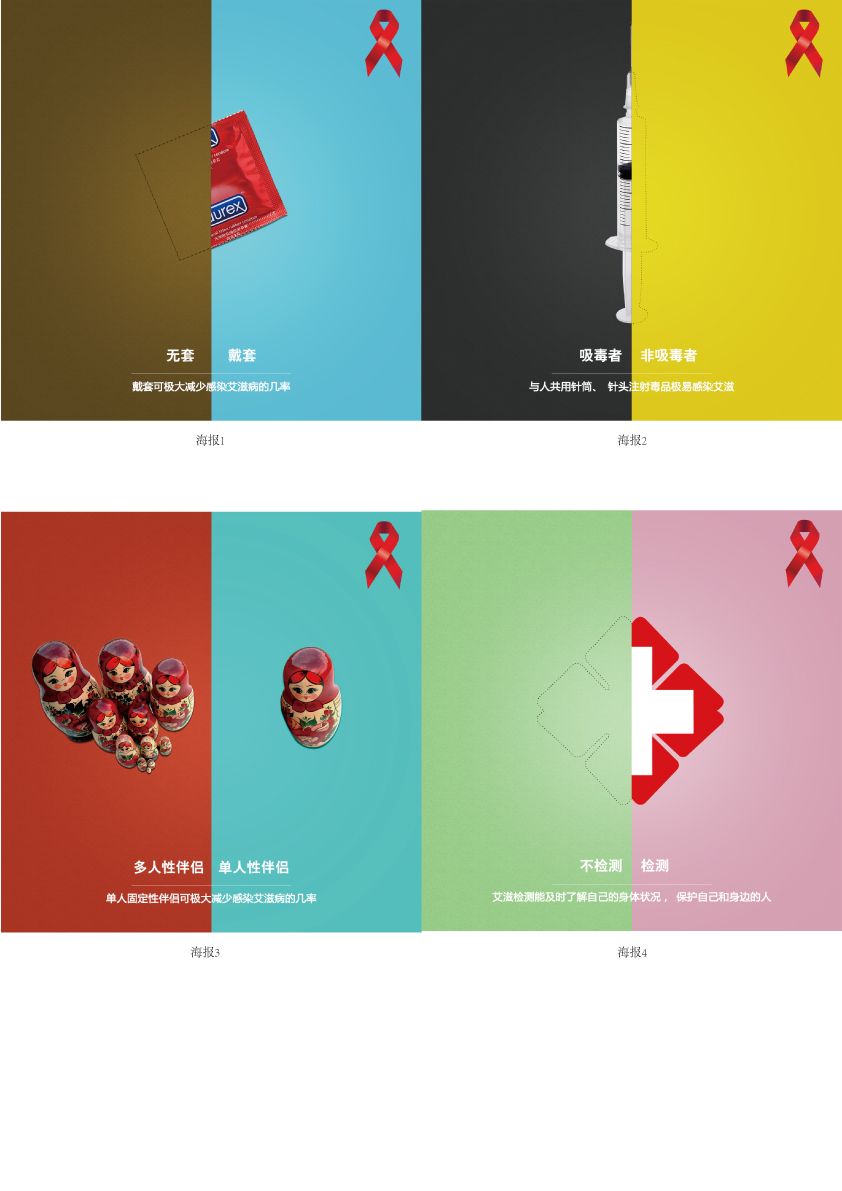

Supplement: Supplementary file 3 — Additional file 3. Intervention materials [file 13063_2020_4860_MOESM3_ESM.zip › Additional file 3/Intervention materials/Images/Image 12.jpg]

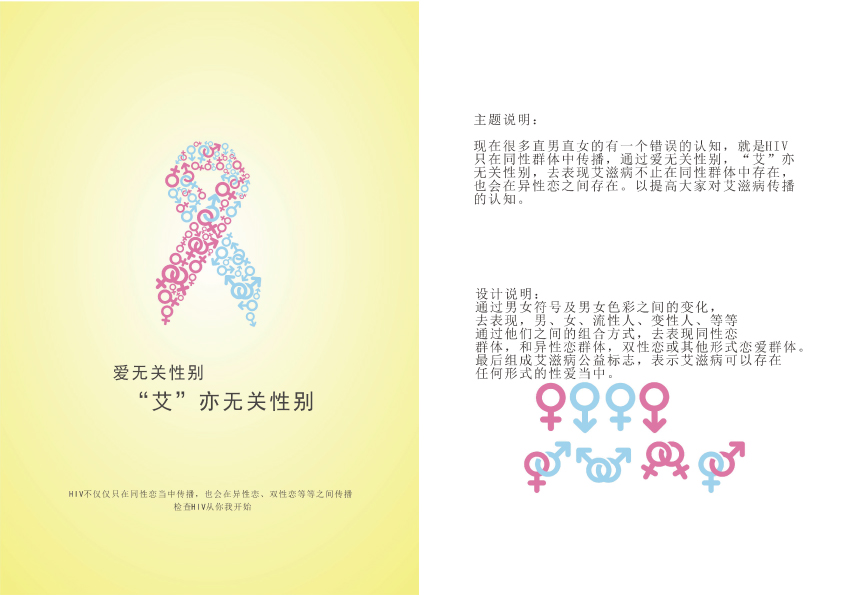

Supplement: Supplementary file 3 — Additional file 3. Intervention materials [file 13063_2020_4860_MOESM3_ESM.zip › Additional file 3/Intervention materials/Images/Image 13.jpg]

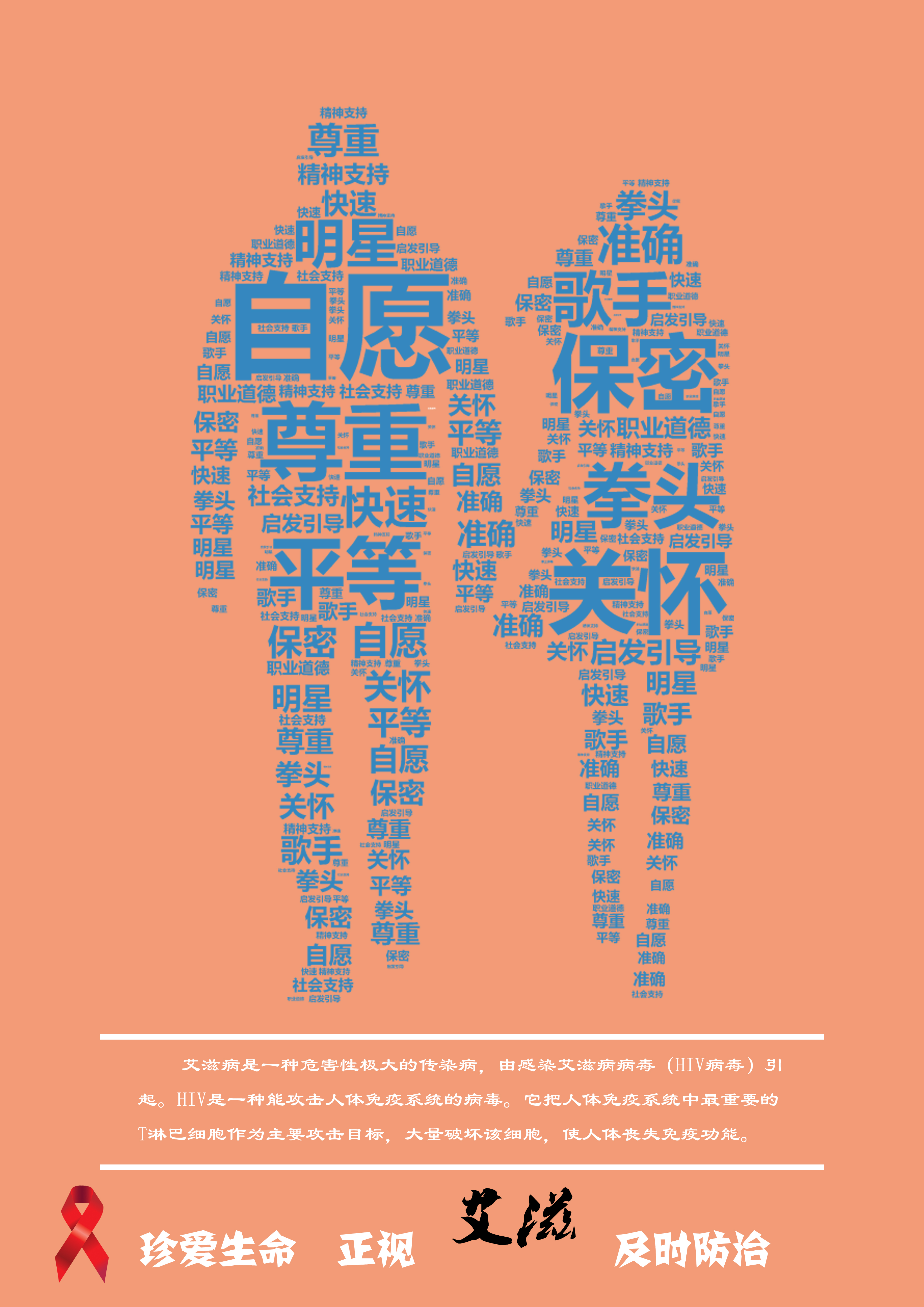

Supplement: Supplementary file 3 — Additional file 3. Intervention materials [file 13063_2020_4860_MOESM3_ESM.zip › Additional file 3/Intervention materials/Images/Image 14.jpg]

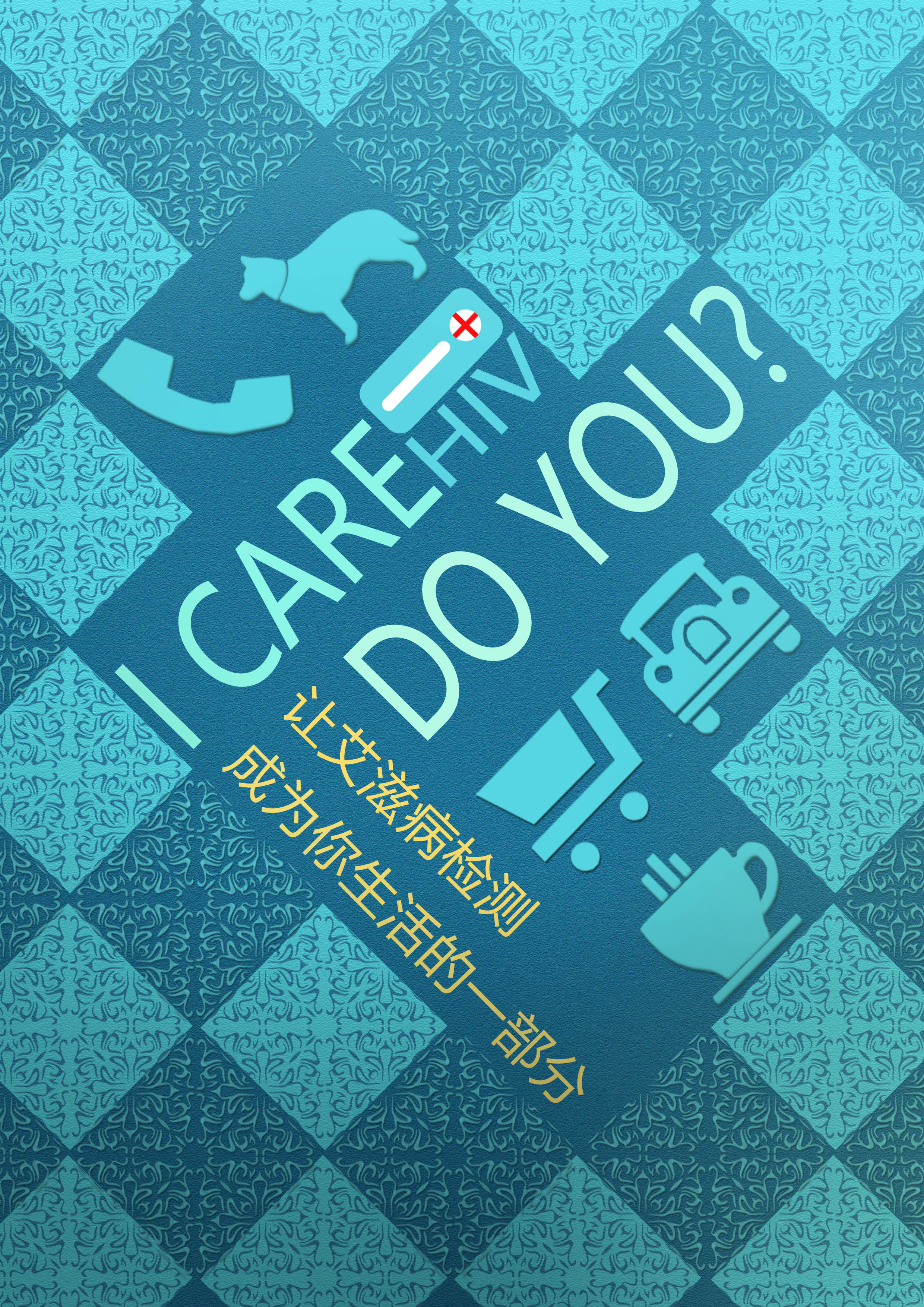

Supplement: Supplementary file 3 — Additional file 3. Intervention materials [file 13063_2020_4860_MOESM3_ESM.zip › Additional file 3/Intervention materials/Images/Image 15.jpg]

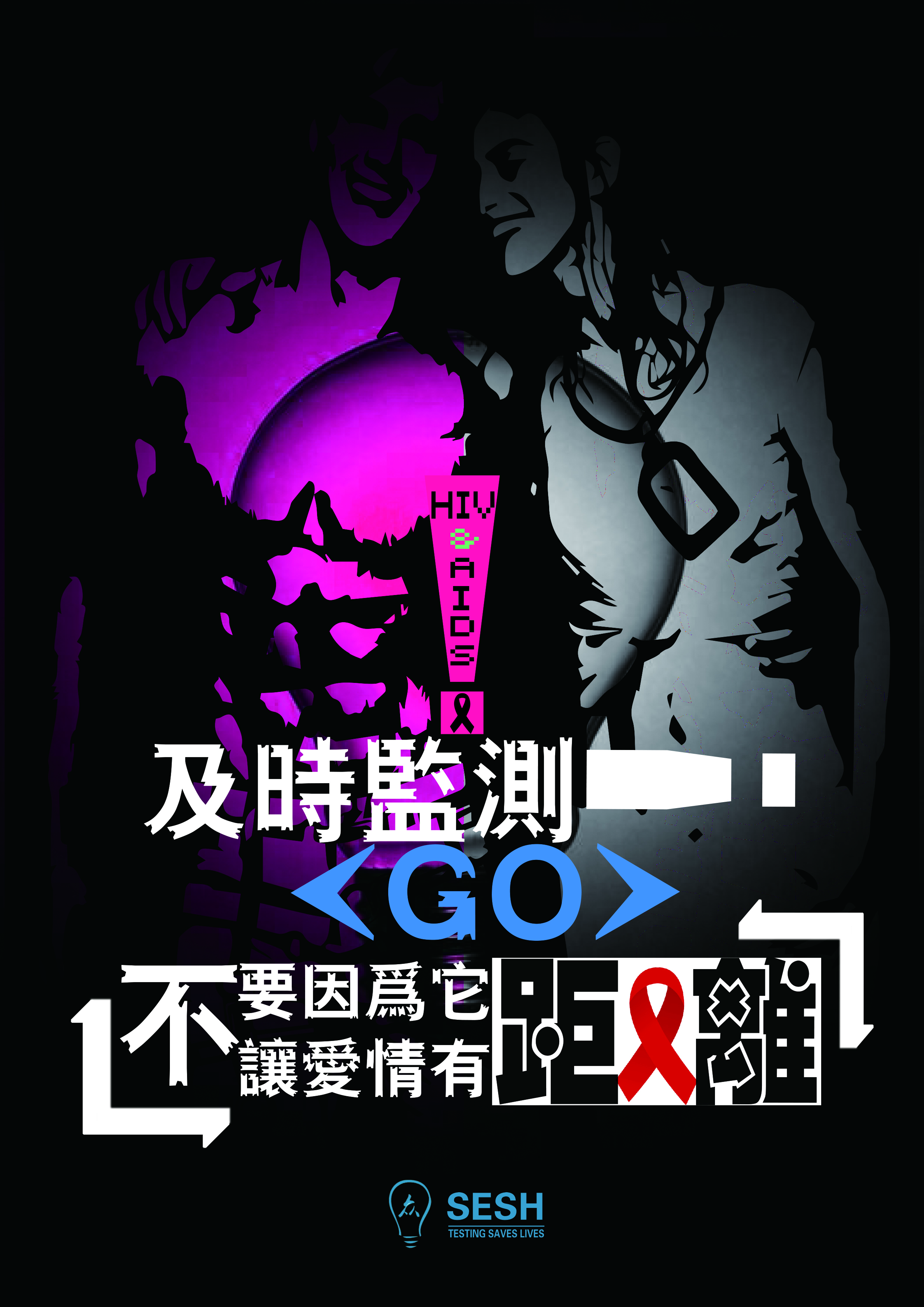

Supplement: Supplementary file 3 — Additional file 3. Intervention materials [file 13063_2020_4860_MOESM3_ESM.zip › Additional file 3/Intervention materials/Images/Image 16.jpg]

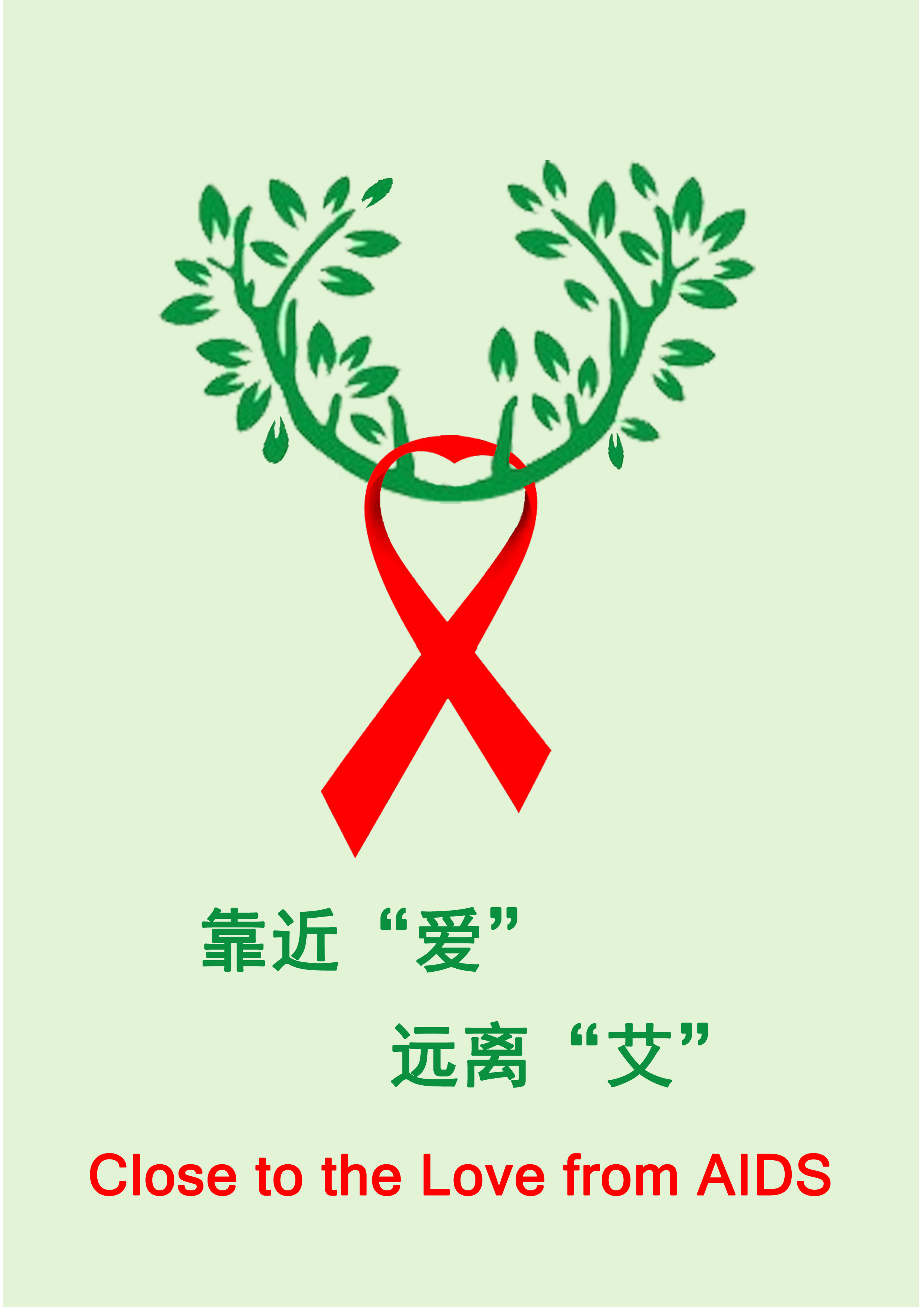

Supplement: Supplementary file 3 — Additional file 3. Intervention materials [file 13063_2020_4860_MOESM3_ESM.zip › Additional file 3/Intervention materials/Images/Image 17.jpg]

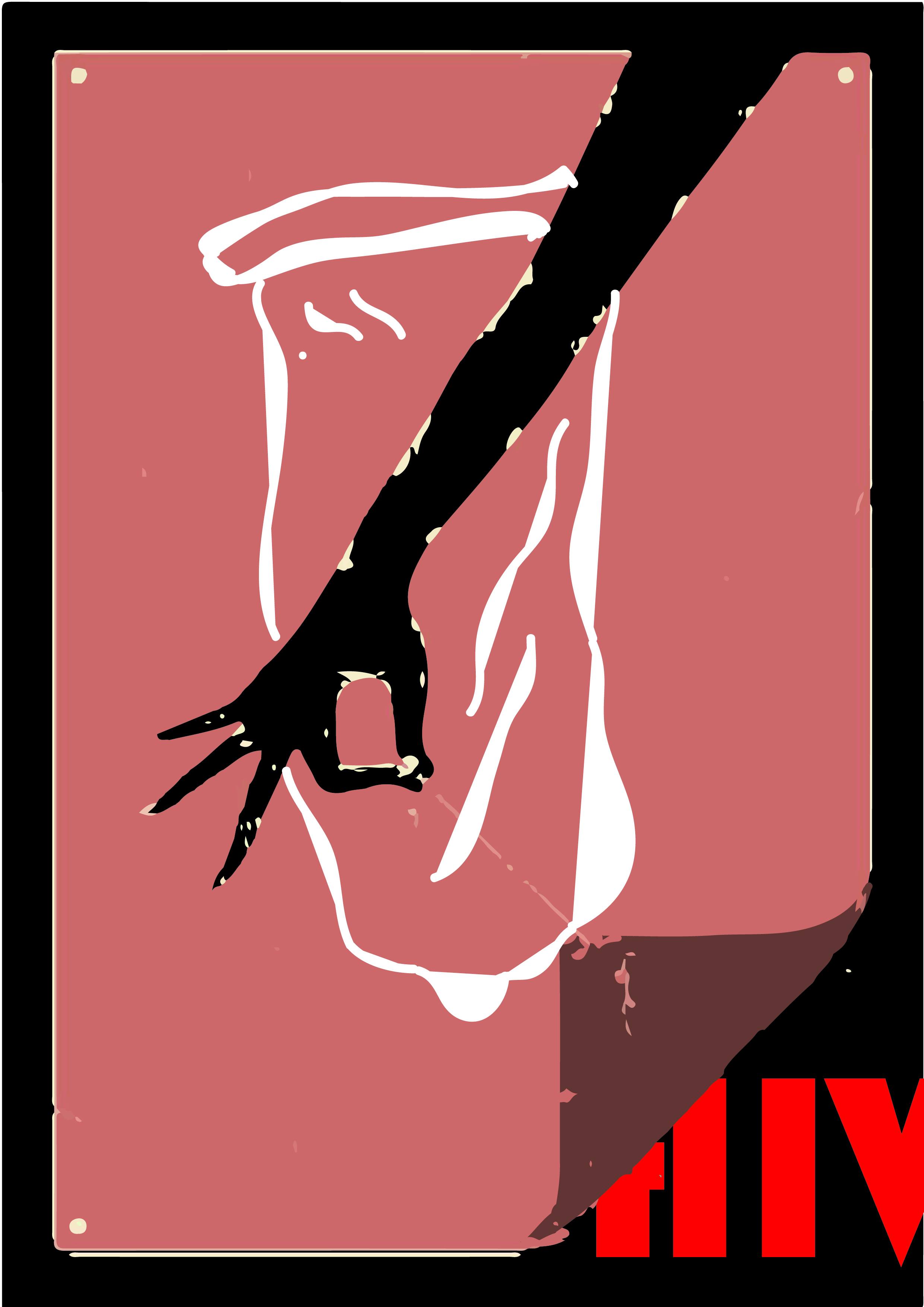

Supplement: Supplementary file 3 — Additional file 3. Intervention materials [file 13063_2020_4860_MOESM3_ESM.zip › Additional file 3/Intervention materials/Images/Image 18.jpg]

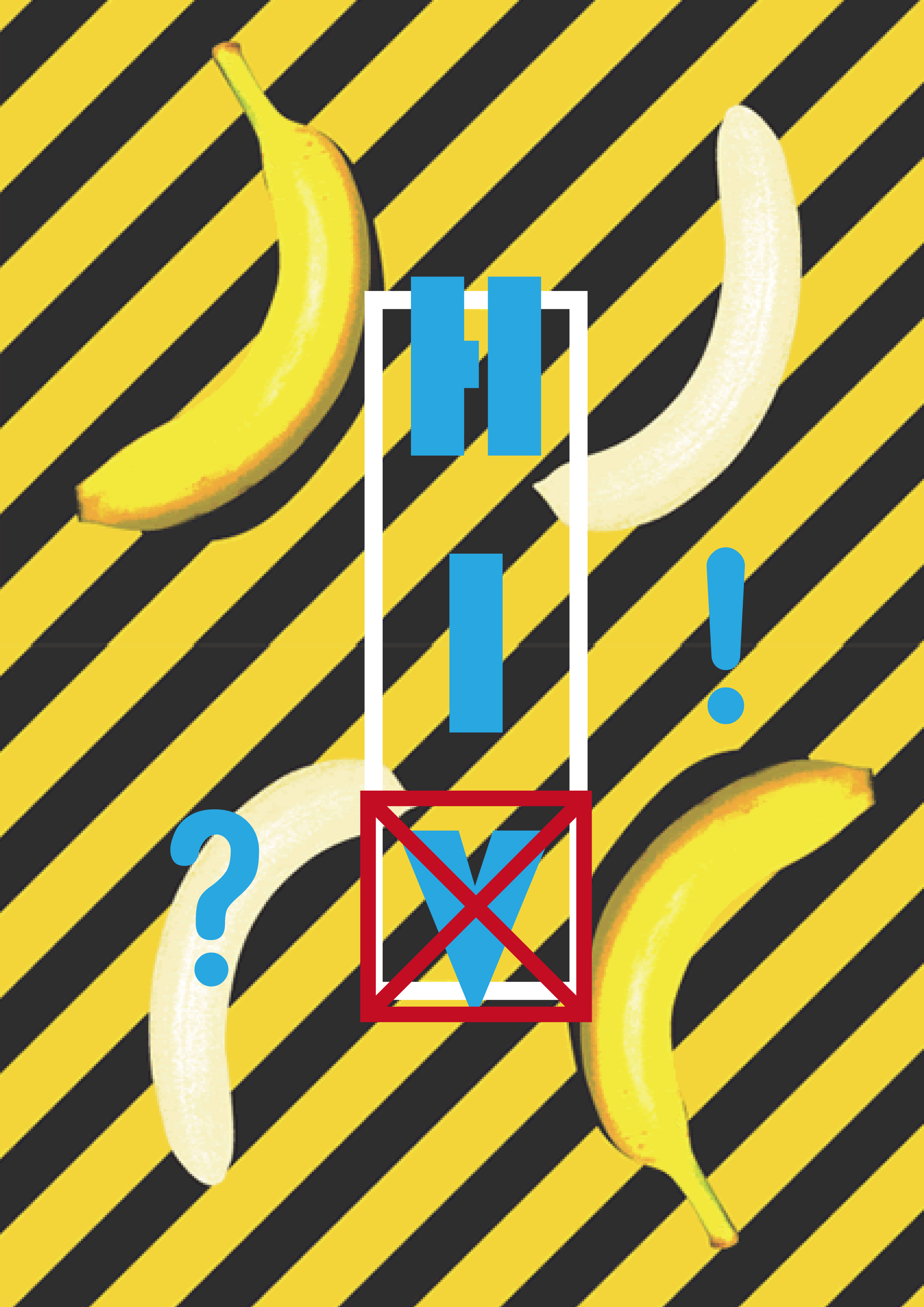

Supplement: Supplementary file 3 — Additional file 3. Intervention materials [file 13063_2020_4860_MOESM3_ESM.zip › Additional file 3/Intervention materials/Images/Image 19.jpg]

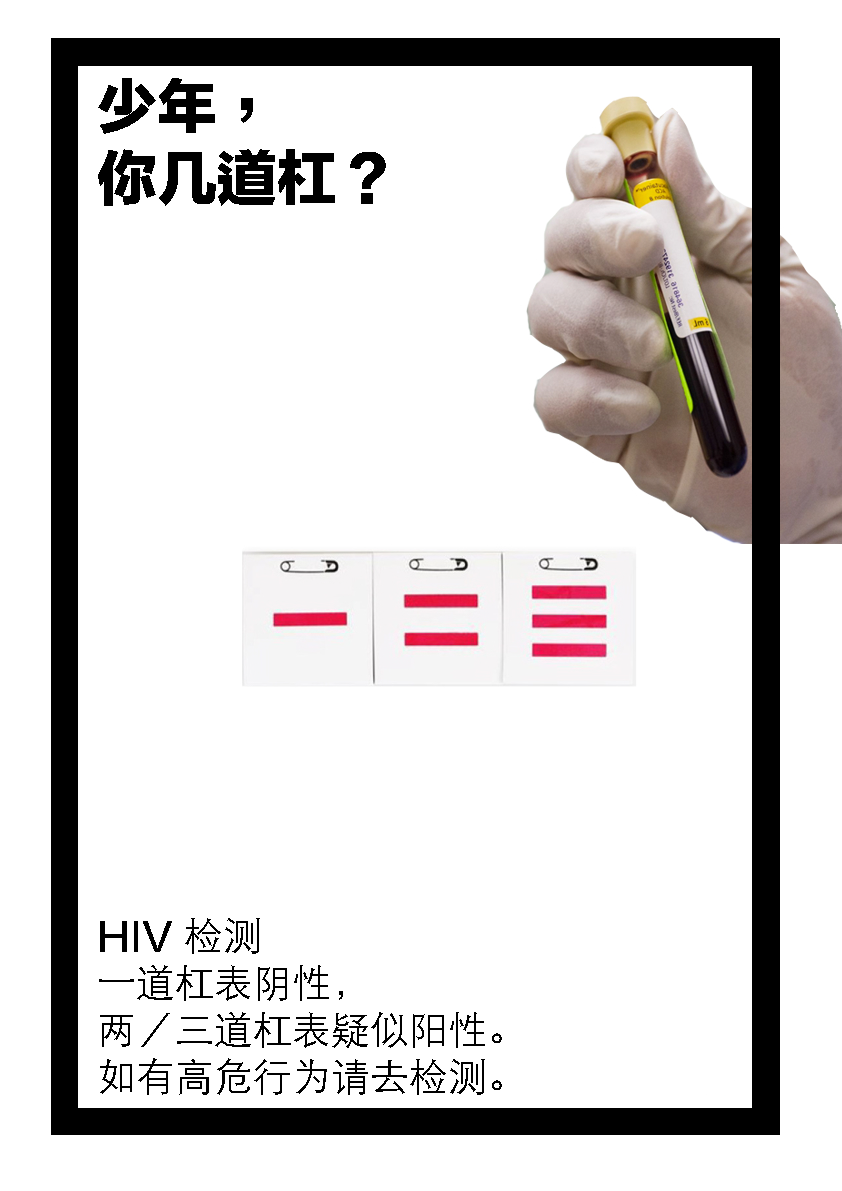

Supplement: Supplementary file 3 — Additional file 3. Intervention materials [file 13063_2020_4860_MOESM3_ESM.zip › Additional file 3/Intervention materials/Images/Image 2.png]

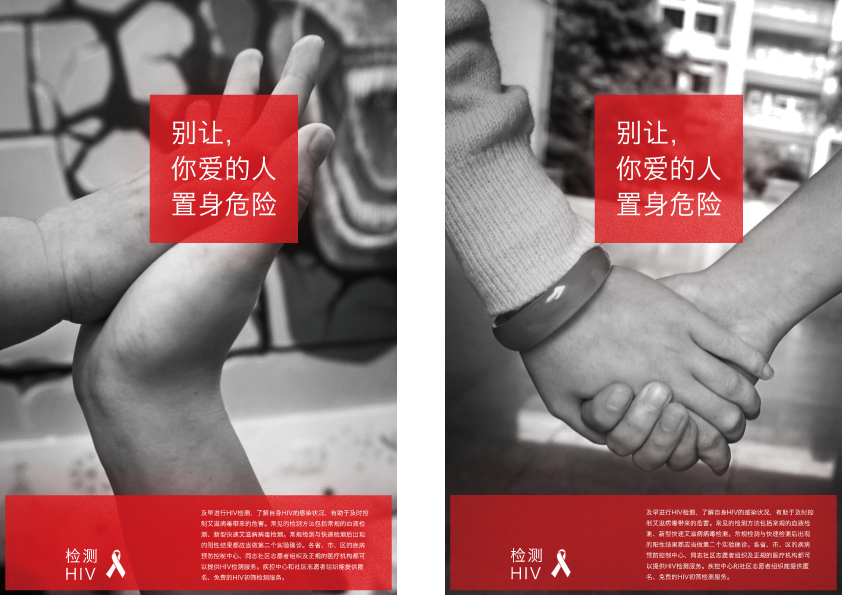

Supplement: Supplementary file 3 — Additional file 3. Intervention materials [file 13063_2020_4860_MOESM3_ESM.zip › Additional file 3/Intervention materials/Images/Image 20.jpg]

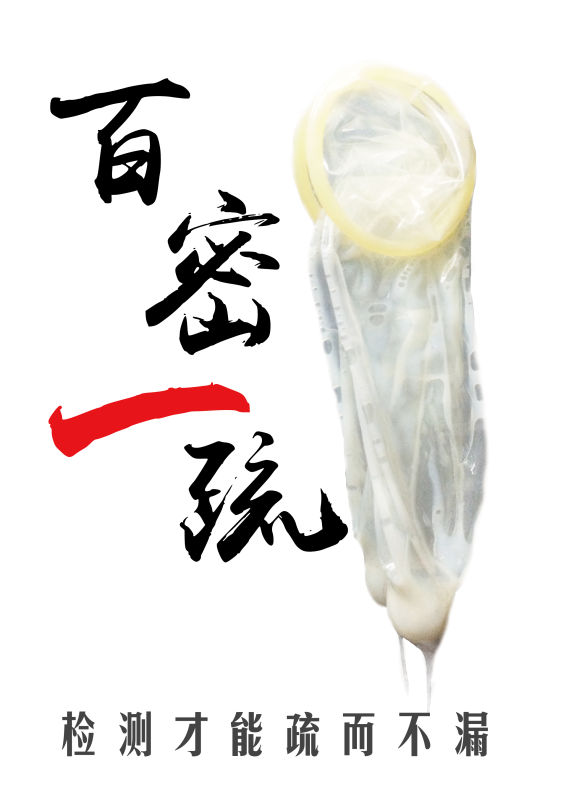

Supplement: Supplementary file 3 — Additional file 3. Intervention materials [file 13063_2020_4860_MOESM3_ESM.zip › Additional file 3/Intervention materials/Images/Image 21.jpeg]

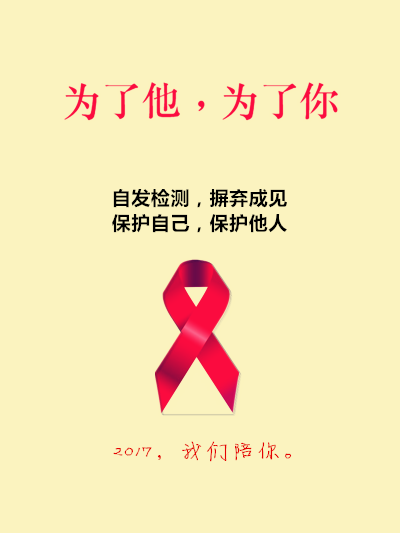

Supplement: Supplementary file 3 — Additional file 3. Intervention materials [file 13063_2020_4860_MOESM3_ESM.zip › Additional file 3/Intervention materials/Images/Image 22.png]

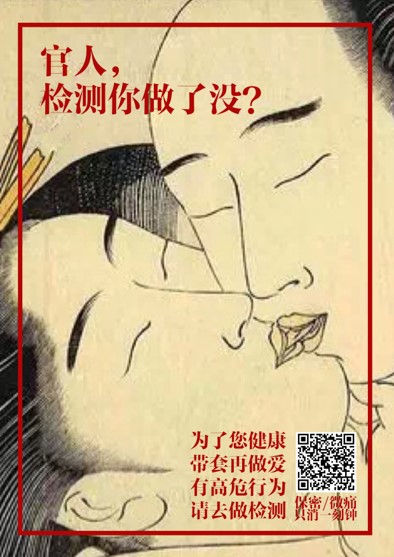

Supplement: Supplementary file 3 — Additional file 3. Intervention materials [file 13063_2020_4860_MOESM3_ESM.zip › Additional file 3/Intervention materials/Images/Image 23.jpg]

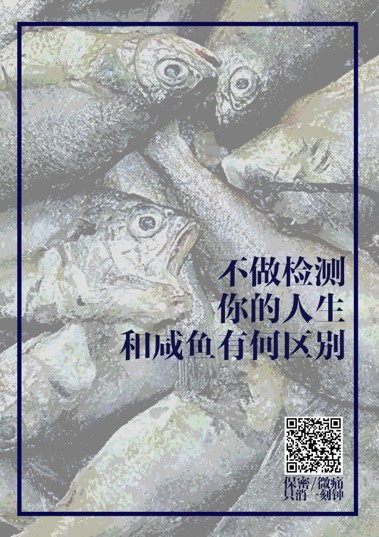

Supplement: Supplementary file 3 — Additional file 3. Intervention materials [file 13063_2020_4860_MOESM3_ESM.zip › Additional file 3/Intervention materials/Images/Image 24.jpg]

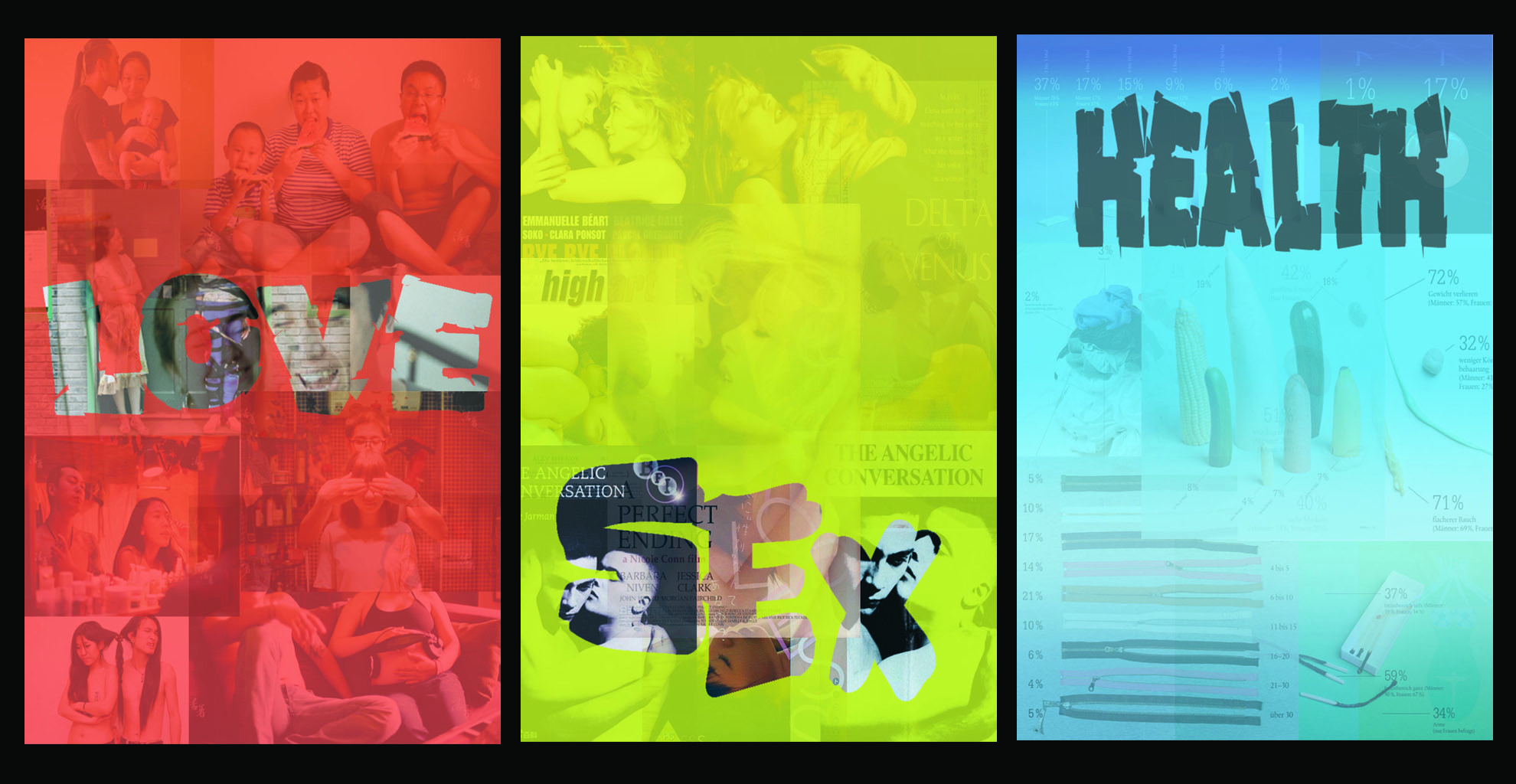

Supplement: Supplementary file 3 — Additional file 3. Intervention materials [file 13063_2020_4860_MOESM3_ESM.zip › Additional file 3/Intervention materials/Images/Image 3.jpg]

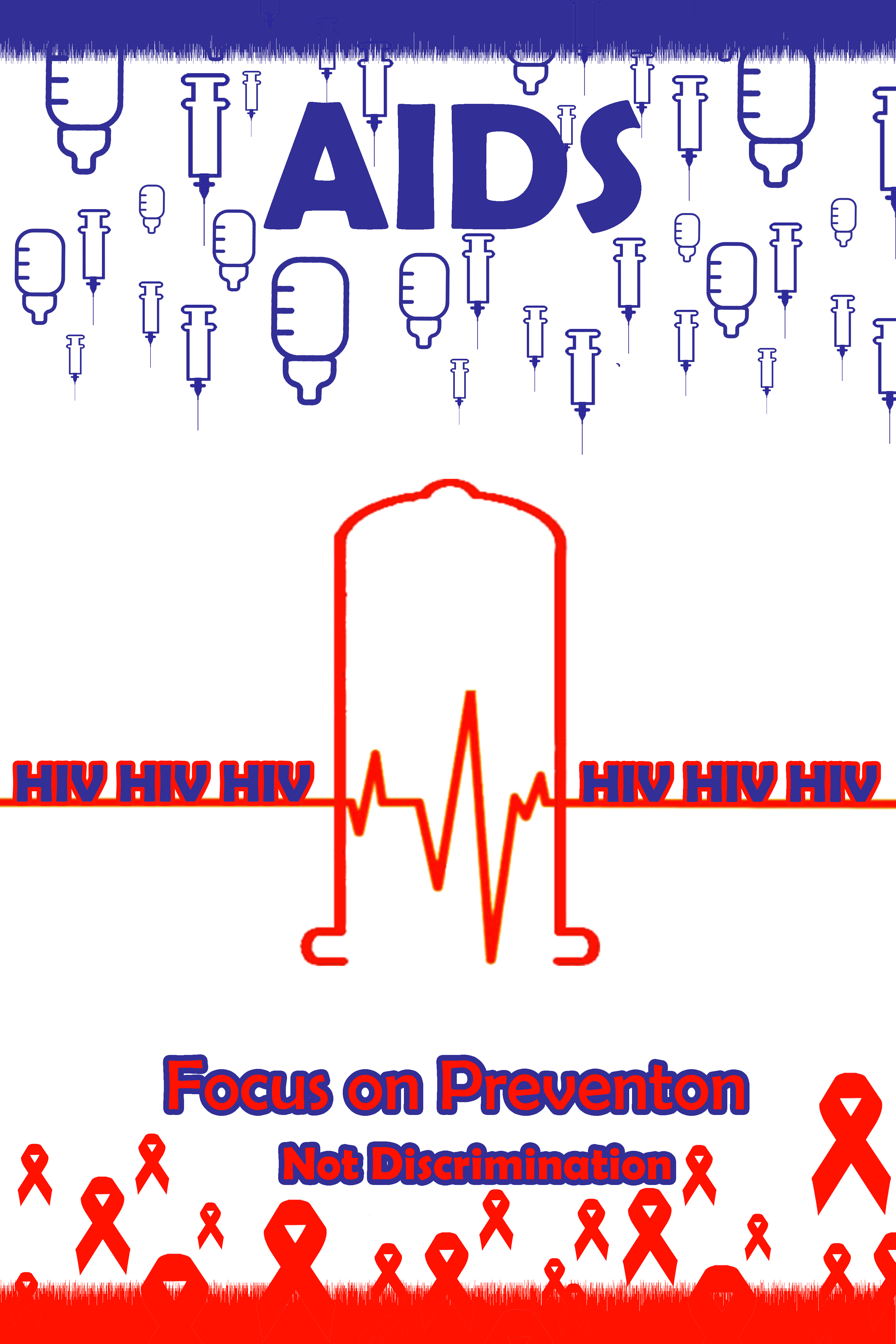

Supplement: Supplementary file 3 — Additional file 3. Intervention materials [file 13063_2020_4860_MOESM3_ESM.zip › Additional file 3/Intervention materials/Images/Image 4.jpg]

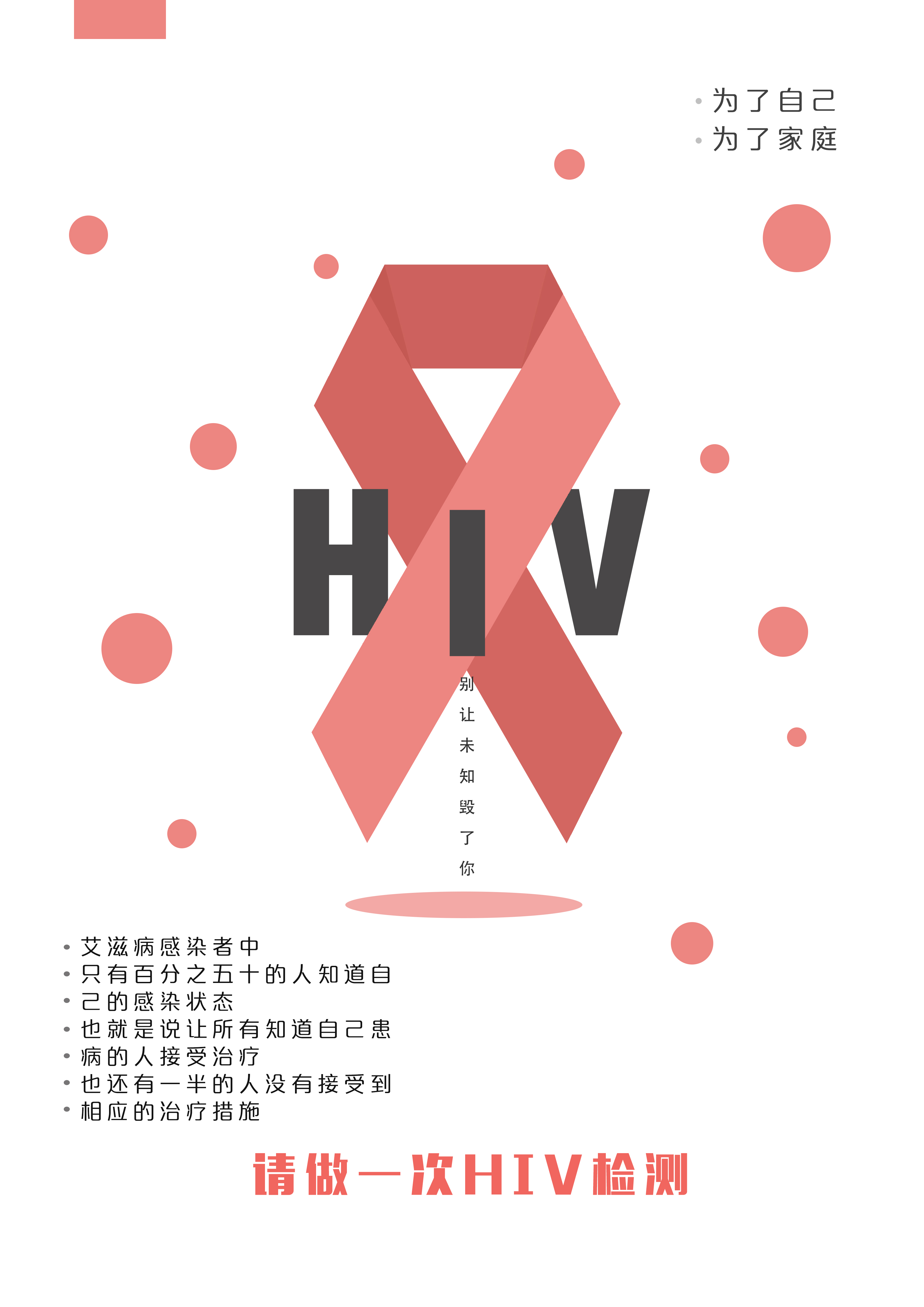

Supplement: Supplementary file 3 — Additional file 3. Intervention materials [file 13063_2020_4860_MOESM3_ESM.zip › Additional file 3/Intervention materials/Images/Image 5.jpg]

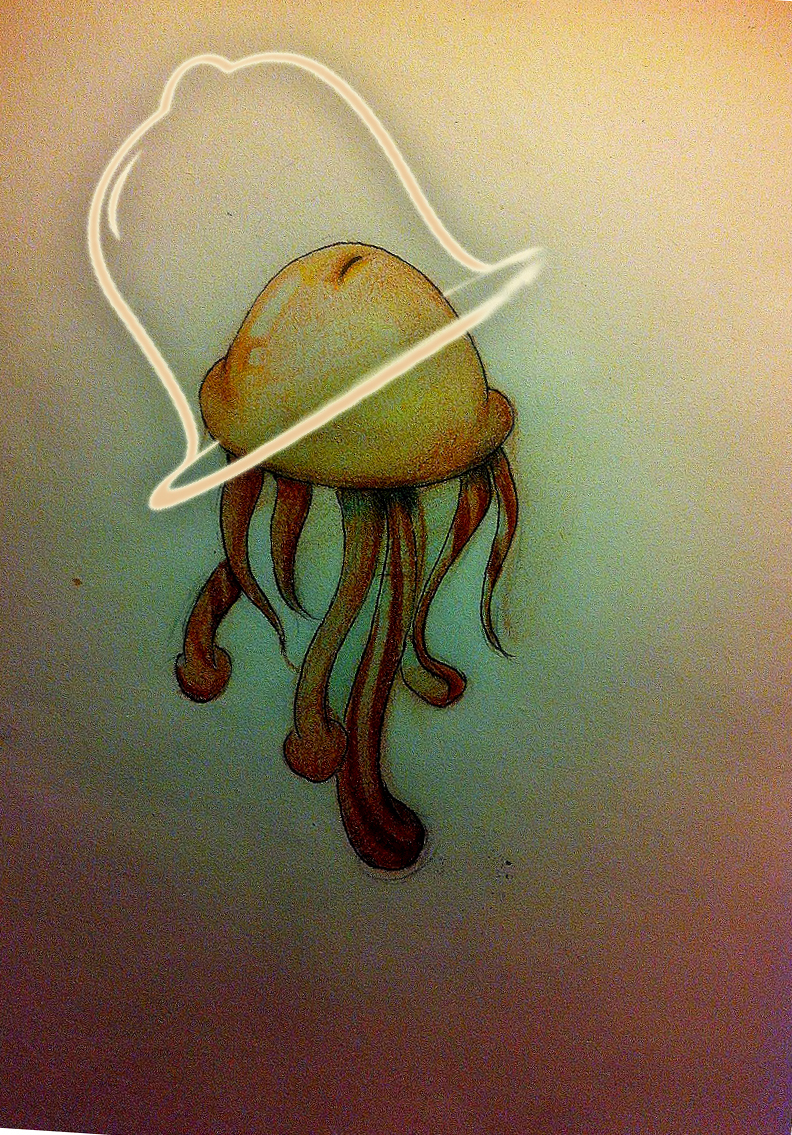

Supplement: Supplementary file 3 — Additional file 3. Intervention materials [file 13063_2020_4860_MOESM3_ESM.zip › Additional file 3/Intervention materials/Images/Image 6.jpg]

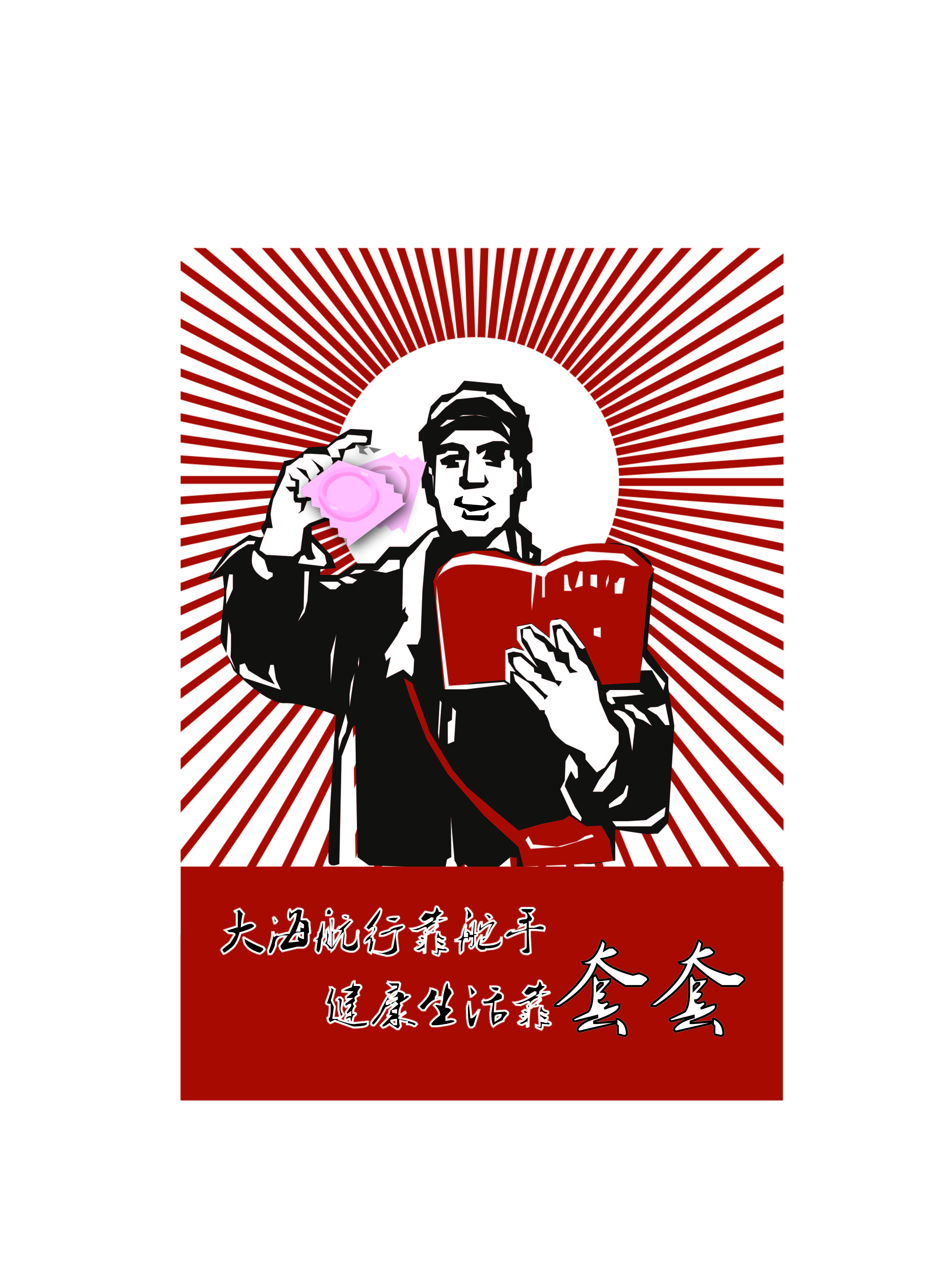

Supplement: Supplementary file 3 — Additional file 3. Intervention materials [file 13063_2020_4860_MOESM3_ESM.zip › Additional file 3/Intervention materials/Images/Image 7.jpg]

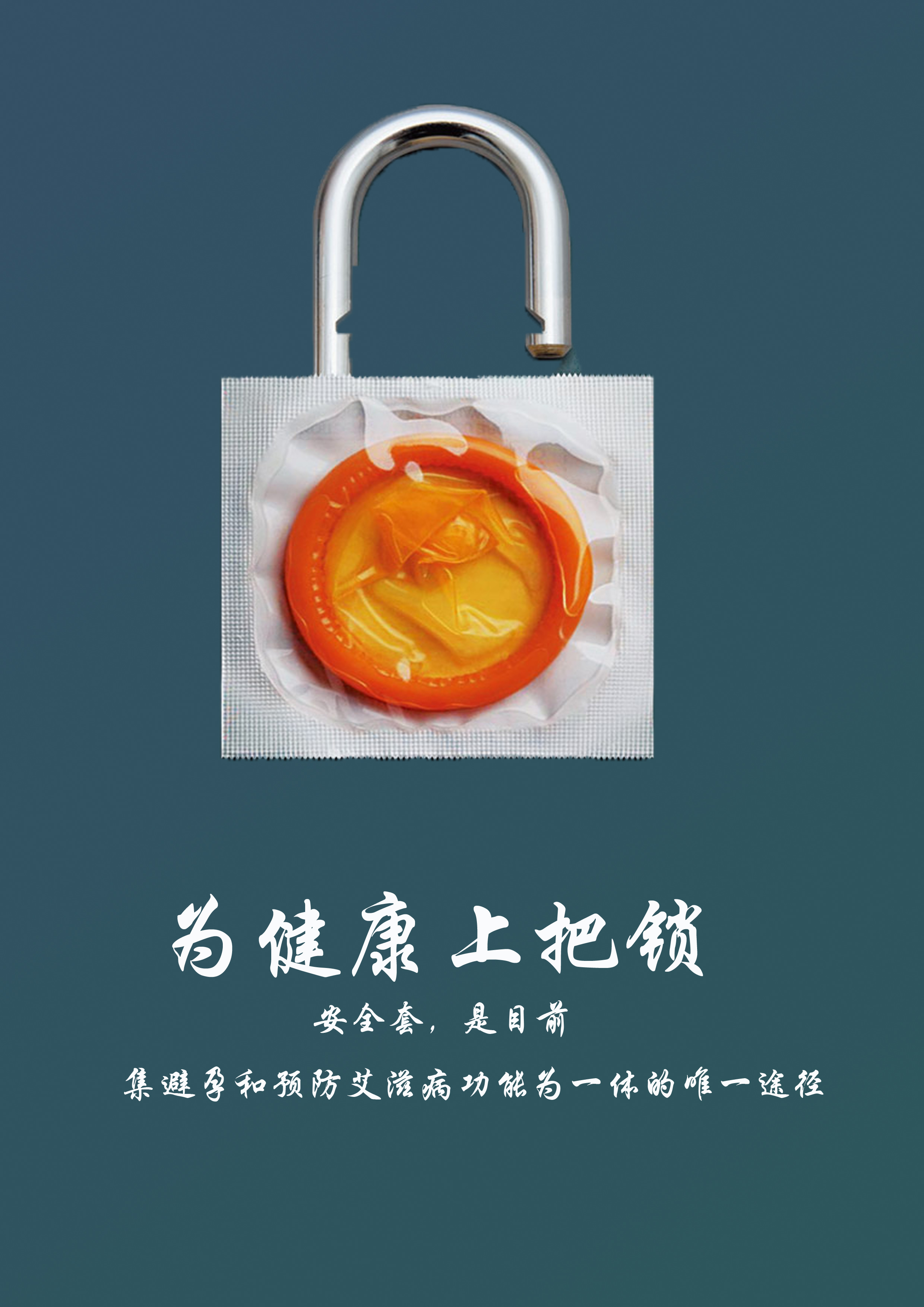

Supplement: Supplementary file 3 — Additional file 3. Intervention materials [file 13063_2020_4860_MOESM3_ESM.zip › Additional file 3/Intervention materials/Images/Image 8.jpg]

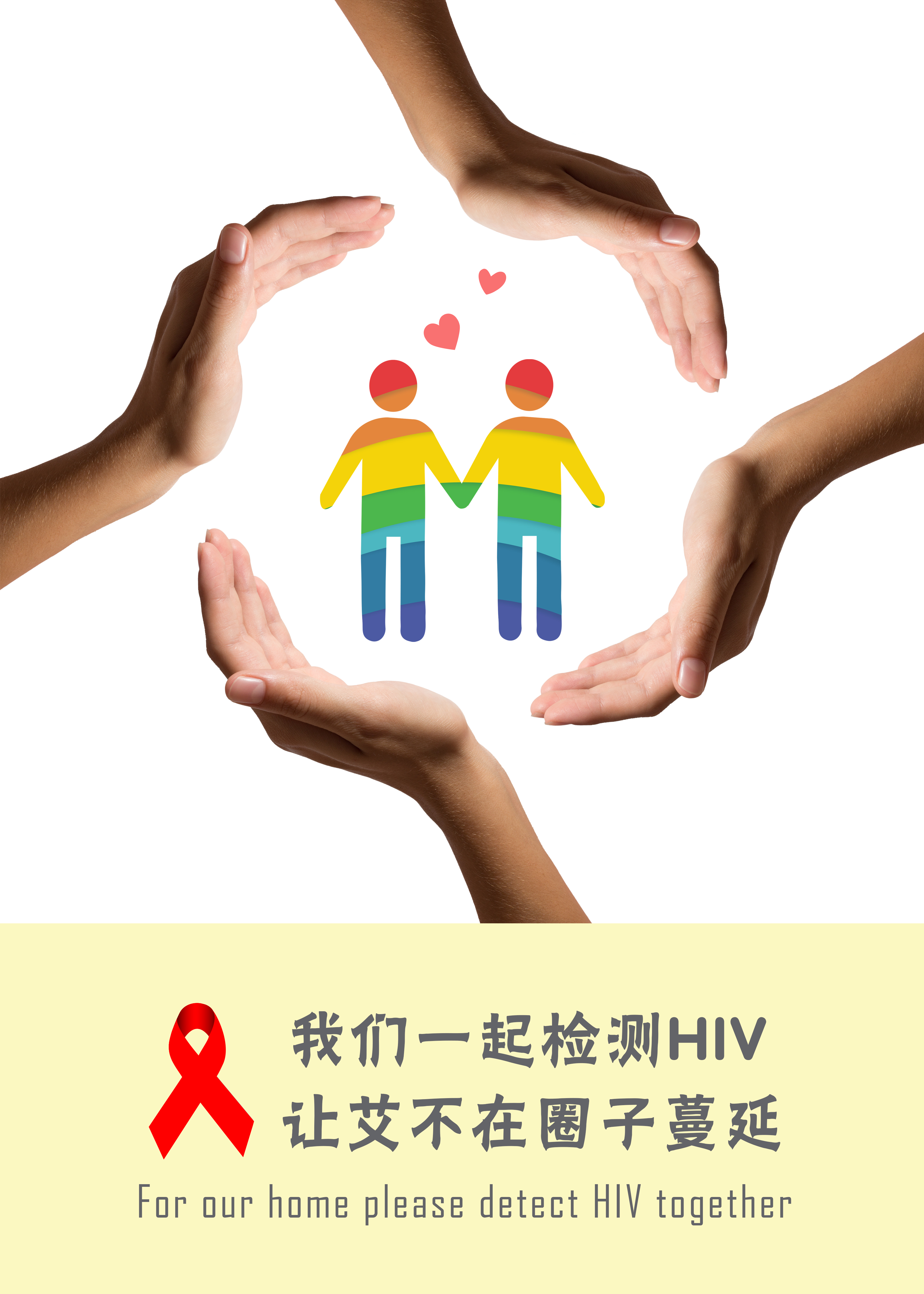

Supplement: Supplementary file 3 — Additional file 3. Intervention materials [file 13063_2020_4860_MOESM3_ESM.zip › Additional file 3/Intervention materials/Images/Image 9.jpg]
