# Supplementary material for: Digital crowdsourced intervention to promote HIV testing among MSM in China: study protocol for a cluster randomized controlled trial
Source: Trials. 2020 Nov 17;21:931. doi: 10.1186/s13063-020-04860-8 (PMC7673095; doi:10.1186/s13063-020-04860-8)
Supplement: Supplementary file 4 — Additional file 4. Table for secondary outcomes [file 13063_2020_4860_MOESM4_ESM.docx]

**Additional File 4. Table for secondary outcomes**

| **Domain** | **Measurement** | **Metric** | **Method of aggregation** | **Main time point** |
| --- | --- | --- | --- | --- |
| *HIV testing* | self-report | between groups | proportion | after 12 months |
| *Condom use* | self-report | between groups | proportion | after 12 months |
| *HIV testing social norms* | self-report | between groups | mean score | after 12 months |
| *HIV testing self-efficacy* | self-report | between groups | mean score | after 12 months |
| *HIV self-testing (self-report)* | self-report | between groups | proportion | after 12 months |
| *HIV self-testing (confirmed)* | pictures of  self-test results  from participants | between groups | proportion | after 12 months |
| *Anticipated*  *HIV stigma* | self-report | between groups | mean score | after 12 months |
| *Syphilis testing* | self-report | between groups | proportion | after 12 months |
| *Mobile app engagement* | self-report | between groups | proportion | after 12 months |
